# Copper(II) Complexes Containing Natural Flavonoid Pomiferin Show Considerable In Vitro Cytotoxicity and Anti-inflammatory Effects

**DOI:** 10.3390/ijms22147626

**Published:** 2021-07-16

**Authors:** Ján Vančo, Zdeněk Trávníček, Jan Hošek, Tomáš Malina, Zdeněk Dvořák

**Affiliations:** 1 Regional Centre of Advanced Technologies and Materials, Czech Advanced Technology and Research Institute (CATRIN), Palacký University, Šlechtitelů 27, CZ-783 71 Olomouc, Czech Republic; jan.vanco@upol.cz (J.V.); jan.hosek@upol.cz (J.H.); tomas.malina@upol.cz (T.M.); 2 Department of Cell Biology and Genetics, Faculty of Science, Palacký University, Šlechtitelů 27, CZ-783 71 Olomouc, Czech Republic; zdenek.dvorak@upol.cz

**Keywords:** copper(II) complexes, pomiferin, in vitro cytotoxicity, cell cycle, ROS, inflammation, nuclease activity

## Abstract

A series of new heteroleptic copper(II) complexes of the composition [Cu(L)(bpy)]NO_3_·2MeOH (**1**), [Cu(L)(dimebpy)]NO_3_·2H_2_O (**2**), [Cu(L)(phen)]NO_3_·2MeOH (**3**), [Cu(L)(bphen)]NO_3_·MeOH (**4**), [Cu(L)(dppz)]NO_3_·MeOH (**5**) was prepared, where HL = 3-(3,4-dihydroxyphenyl)-5-hydroxy-8,8-dimethyl-6-(3-methylbut-2-ene-1-yl)-4*H*,8*H*-benzo[1,2-*b*:3,4-*b*′]dipyran-4-one, (pomiferin) and bpy = 2,2′-bipyridine, dimebpy = 4,4′-dimethyl-2,2′-bipyridine, phen = 1,10-phenanthroline, bphen = 4,7-diphenyl-1,10-phenanthroline, and dppz = dipyrido[3,2-*a*:2′,3′-*c*]phenazine. The complexes were characterized using elemental analysis, infrared and U*V/V*is spectroscopies, mass spectrometry, thermal analysis and conductivity measurements. The in vitro cytotoxicity, screened against eight human cancer cell lines (breast adenocarcinoma (MCF-7), osteosarcoma (HOS), lung adenocarcinoma (A549), prostate adenocarcinoma (PC-3), ovarian carcinoma (A2780), cisplatin-resistant ovarian carcinoma (A2780R), colorectal adenocarcinoma (Caco-2) and monocytic leukemia (THP-1), revealed the complexes as effective antiproliferative agents, with the IC_50_ values of 2.2–13.0 μM for the best performing complexes 3 and 5. All the complexes 1–5 showed the best activity against the A2780R cells (IC_50_ = 2.2–6.6 μM), and moreover, the complexes demonstrated relatively low toxicity on healthy human hepatocytes, with IC_50_ > 100 μM. The complexes were evaluated by the Annexin V/propidium iodide apoptosis assay, induction of cell cycle modifications in A2780 cells, production of reactive oxygen species (ROS), perturbation of mitochondrial membrane potential, inhibition of apoptosis and inflammation-related signaling pathways (NF-κB/AP-1 activity, NF-κB translocation, TNF-α secretion), and tested for nuclease mimicking activity. The obtained results revealed the corresponding complexes to be effective antiproliferative and anti-inflammatory agents.

## 1. Introduction

Cisplatin and its derivatives represent a group of very successful cancer chemotherapeutic drugs; however, the utilization of these platinum-based complexes is complicated by several adverse effects (e.g., neurotoxicity, nephrotoxicity and emetogenesis) and intrinsic and/or acquired resistance phenomena [1,2]. These drawbacks motivate scientists to find a new generation of other transition metal complexes with an improved anticancer effect and simultaneously with suppressed negative side-effects and toxicity towards healthy cells. It seems that one of the promising candidates to fulfil these requirements could be associated with coordination compounds containing copper as a central metal atom [3]. Copper(II) complexes, involving various ligands, are extensively studied nowadays for their promising biological features, such as antiproliferative, antioxidant, antifungal or antibacterial effects. Moreover, copper-containing complexes or materials also behave as bearers of high thermal and electrical conductivity, or catalytic and sensory features. As examples, the following papers could be mentioned to show the current strives on the design of copper(II) complexes showing some of the above mentioned properties, mainly the antiproliferative one [4,5,6,7,8,9,10]. To date, one of the most intensively studied groups of copper(II) complexes in terms of their possible clinical use as anticancer agents is the copper(II) complexes called Casiopeínas^®^, whose composition can be expressed by the formulas [Cu(*N*–*N*)(*N*–*O*)]^+^ and/or [Cu(*N*–*N*)(*O*–*O*)]^+^, where (*N*–*N*) represents an aromatic heterocyclic diimine, such as 2,2′-bipyridine (bpy) or 1,10-phenanthroline (phen), (*N*–*O*) and (*O*–*O*) stands for an amino acid, and acetylacetone, respectively [11]. These complexes were firstly prepared by Lena Ruiz-Azuara and colleagues in the 1990s [12]. Casiopeínas^®^ have proven their significant anticancer activity both in preclinical in vitro and in vivo studies and currently, some of them entered the Phase I of the clinical trials [13].

**Figure 1:**
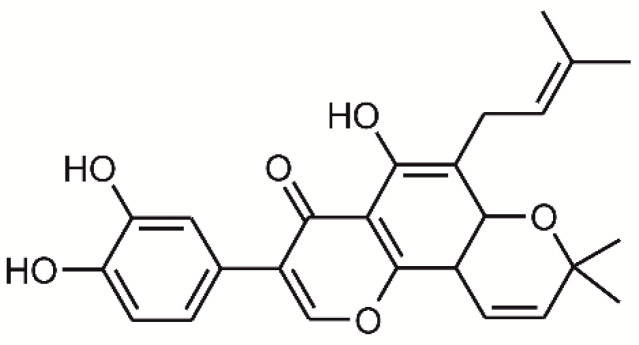
Structural formula of a naturally occurring prenylated flavonoid pomiferin, 3-(3,4-dihydroxyphenyl)-5-hydroxy-8,8-dimethyl-6-(3-methylbut-2-ene-1-yl)-4*H*,8*H*-benzo[1,2-*b*:3,4-*b′*]dipyran-4-one.

These positive findings inspired many scientists to study copper(II) complexes having the N_2_O_2_ and N_3_O donor sets and containing the bpy and phen ligands or their derivatives more deeply. In accordance with these outcomes, we have focused on the study of heteroleptic copper(II) complexes containing heterocyclic diimine *N–N* donor ligands together with quinolinone derivatives as *O–O* donor ligands of the composition [Cu(qui)(phen)]Y·*x*H_2_O, where Hqui = 2-phenyl-3-hydroxy-4(1*H*)-quinolinone, Y = NO_3_ or BF_4_, and phen = 1,10-phenanthroline and its derivatives. These complexes revealed significant in vitro cytotoxicity against human cancer cell lines, such as A549 lung carcinoma, HeLa cervix epithelioid carcinoma, G361 melanoma cells, A2780 ovarian carcinoma, A2780cis cisplatin-resistant ovarian carcinoma, LNCaP androgen-sensitive prostate adenocarcinoma and THP-1 monocytic leukemia, with the best IC_50_ reaching the values of 0.36–0.73 μM against A2780 and A2780R [14] and against HOS lung osteosarcoma, MCF-7 breast adenocarcinoma as published for complexes of [Cu(qui)(phen)]NO_3_·*x*H_2_O [15], and for complexes of [Cu(qui)(phen)]BF_4_·*x*H_2_O [16]. Very promising results were also found in the case of similar 1,10-phenanthroline complexes having a composition of [Cu(quin)(phen)]NO_3_·*y*H_2_O, where Hquin stands for variously *N*-substituted derivatives of 2-(4-amino-3,5-dichlorophenyl)-3-hydroxy-4(1*H*)-quinolinone-7-carboxamide, which showed significant in vitro cytotoxicity of IC_50_ ≈ 1–7 µM on HOS, MCF-7, G361, HeLa, A2780 and A2780R [17]. Moreover, the second generation of mixed ligand copper(II)-1,10-phenathroline complexes, containing derivatives of 2′-hydroxychalcone-{(*E*)-1-(2′-hydroxyphenyl)-3-phenylprop-2-en-1-one}, Hchal, of the composition [Cu(chal)(phen)]NO_3_, which we reported previously [18], also revealed substantial cytotoxicity on a panel of human cancer cell lines G361, HeLa, MCF-7, HOS, A549, HepG2, PC-3, 22Rv1, A2780 and A2780R, with the IC_50_ values ranging from 1.4 to 15.7 µM. These findings motivated us further to select such a bidentate *O*,*O*-donor ligand, which will bear its own antiproliferative activity against human cancer cells, and that is why we focused on a natural flavonoid pomiferin, 3-(3,4-dihydroxyphenyl)-5-hydroxy-8,8-dimethyl-6-(3-methylbut-2-ene-1-yl)-4*H*,8*H*-benzo [1,2-*b*:3,4-*b′*]dipyran-4-one, (see Figure 1) occurring as a secondary metabolite for example in *Maclura pomifera* (Rafin.) Schneid (*Moraceae*) [19,20]. Pomiferin belongs to a group of prenylated isoflavones known to possess a broad spectrum of various biological activities both in vitro and in vivo. Some flavonoids, including pomiferin, are able to chelate metal ions [21], and therefore, many transition metal complexes of flavonoids were prepared to date [22]; however, none of the prenylated isoflavones. Flavonoid metal complexes usually possess superior biological activities to parental molecules, including cytotoxic, anti-inflammatory and oxidative-reduction effects [22]. Pomiferin showed strong antioxidant effects in several biochemical models of oxidative stress [23,24], and also in vivo animal models [25,26]. On the other hand, it showed also a mild pro-oxidant effect in cell-based models [27]. Several studies also confirmed an inhibitory effect of pomiferin on enzymes, such as histone deacetylase (HDAC) [28] and acetylcholine esterase (AChE) [29] and its interaction with cannabidiol receptors CB1 and CB2 [20]. Pomiferin is able to protect indomethacin-induced gastric ulcer formation [30] and has antiproliferative effects against cancer cells, but not against non-transformed cells [28,31]. An anti-inflammatory potential was also ascribed to pomiferin [27,32].

In addition, the anticancer activity of pomiferin against human cancer cell lines of ACHN renal carcinoma, NCI-H23 lung adenocarcinoma, PC-3 prostate carcinoma, MDA-MB-231 breast carcinoma, LOX-IMVI melanoma, colon carcinoma after 24 h of incubation with GI_50_ values in the range of 1.32–13.32 µM [28], and also against HuCCA-1 cholangiocarcinoma, KB epithelioid carcinoma, HeLa cervical carcinoma and T47D breast carcinoma after 48 h incubation with ED_50_ values in the range of 0.6–24 µM [33] was described in several papers. On the other hand, the paper of Ribaudo G. et al. (2019) [34] disputes the cytotoxicity of these compounds against both human cancer and healthy cells.

As for the copper(II) complexes with simple isoflavonoids as ligands, there has been described just one copper(II) complex containing genistein (HL) with the general formula [Cu(L)_2_], having the systemic name of bis[3-(4-hydroxyphenyl)-7-hydroxy-4*H*-benzopyran-4-one-5-olato-κO^2^] copper(II) [35]. The cytotoxicity of this complex against human cancer cell lines has been tested after 72 h incubation, specifically against 518A2 melanoma cells (with IC_50_ = 10.9 ± 1.4 µM) [35], Panc-1 pancreas carcinoma cells (IC_50_ = 16.9 ± 0.8 µM), MCF-7/Topo breast adenocarcinoma cells IC_50_ = 15.0 ± 1.5 µM), KB-V1/Vbl cervix carcinoma cells (IC_50_ = 8.0 ± 0.2 µM), DLD-1 colon carcinoma (IC_50_ = 21.2 ± 1.8 µM) and U-87 MG glioblastoma (IC_50_ = 30.2 ± 5.0 µM) [36]. In all the above mentioned cases, except for the KB-V1/Vbl cell line, the cytotoxicity of the studied complex equalled or was significantly lower in comparison with the reference drug cisplatin (IC_50_ values for cisplatin varies in the range of 4.0–32.6 µM on the mentioned cancer cell lines). The toxicity of the studied copper(II) complex against human healthy Ea.Hy 926 endothelial hybrid cells (IC_50_ = 20.1 ± 0.7 µM) was comparable to the level of toxicity of the reference drug cisplatin (IC_50_ = 17.3 ± 1.9 µM) [36].

To extend the above mentioned observations, and with the aim to prepare compounds with significant in vitro cytotoxicity against human cancer cells and preferably with negligible toxicity on healthy cells, a series of new heteroleptic copper(II) complexes containing pomiferin (HL) and bidentate *N*-donor ligands derived from 2,2′-bipyridine (bpy) and 1,10-phenanthroline (phen) was prepared. The complexes of the general formula [Cu(L_1_)(*N–N*)]NO_3_ *x*solv (*x* = 0, 1 or 2; solv = MeOH or H_2_O), were evaluated for their cytotoxic effect on a panel of human cancer cell lines and anti-inflammatory potential in vitro. The mechanisms associated with these biological activities were uncovered using the series of tests, involving the evaluation of cell death induction by the Annexin V/propidium iodide assay, induction of cell cycle modifications in A2780 cells, production of reactive oxygen species (ROS), perturbation of mitochondrial membrane potential, inhibition of inflammation-related signalling pathways (NF-κB/AP-1 activity, NF-κB translocation, TNF-α secretion), and nuclease mimicking activity. The possible utilization of the complexes **1**–**5** in the preparation of drugs for the treatment of tumour diseases forms a part of the patent no. CZ 308 426 B6 granted by the Industrial Property Office of the Czech Republic, and it is also a part of international patent application no. PCT/CZ2020/050050, published under no. WO/2021/018324. Based on the literature search, it can be concluded that the copper(II) complexes, containing pomiferin and bidentately-coordinated *N*-donor ligand, represent a novel and not yet described group of biologically active complexes of copper.

## 2. Results and Discussion

### 2.1. Synthesis and Characterization

**Scheme 1:**
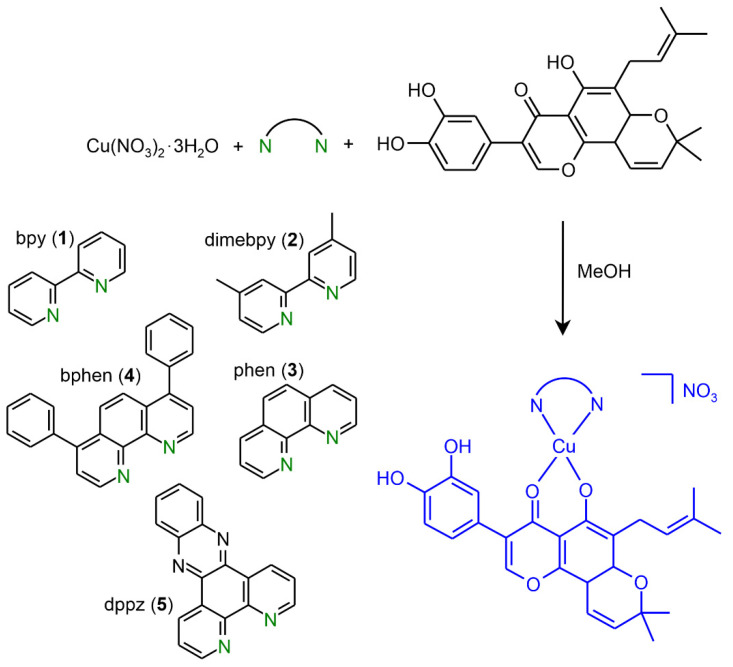
A general pathway leading to the preparation of the complexes **1**–**5**. N: the coordination atoms of the N-donor ligands.

The complexes **1**–**5** were prepared by a general procedure described in detail in the granted Czech patent no. CZ 308 426 B6 and in the international patent application no. PCT/CZ2020/050050 with publication no. WO/2021/018324. Generally speaking, the corresponding bidentate *N,N′*-donor ligand (bpy, dimebpy, phen, bphen and dppz) reacts with Cu(NO_3_)_2_·3H_2_O in MeOH and consequently with pomiferin in the molar ratio of 1:1:1. A general pathway leading to the preparation of complexes **1**–**5** is shown in Scheme 1. The purity and composition of the complexes were determined by means of elemental analysis, infrared and electronic spectroscopies, mass spectrometry, thermal analysis and conductivity measurements. The IR spectra of all the complexes **1**–**5** confirmed the presence of pomiferin, the corresponding *N*-donor ligand and nitrate anion within the molecules [37,38], because the following characteristic absorption peaks were observed in the proximity of the following regions: 3200 ν(OH), 3050 ν(CH)_arom._, 2970 ν(CH)_aliph_, 2920, 1630 and 1580 ν(C–C)_arom._, 1530 ν(C–C)_arom._, 1480, 1420 ν(C–C)_arom_. and/or ν_a_(NO_2_) in an unidentate coordination, 1370, 1300 ν_s_(NO_2_) in an unidentate coordination, 1220 and 1190 ν(C–O), 1150, 1120, 1050 ν(C–O)_MeOH_, 1000 ν(NO), 830, 760 δ(C–H), 720 cm^−1^, for detailed information see Section 3.2. The IR spectra of the complexes **1**–**5** and pomiferin (HL) are shown in Supplementary Materials as Figures S1–S6. The presence of the solvent molecules within the complexes was suggested by the results of elemental analyses and proven in the case of the representative complexes **4** (a weight loss exp./calcd. = 4.08/3.52%) and **5** (a weight loss exp./theor. = 3.75/3.73%) using TG/DSC curves, which confirmed good agreements between the experimental and theoretical values of weight losses, see Supplementary Materials, in Figures S7 and S8, respectively. The U*V/V* is spectra were measured both in the solid state (see Supplementary Materials, Figure S9) and in the solutions (DMF (see Supplementary Materials, Figure S10), MeCN and MeOH) and they pointed out a clear maxima of charge transfer transitions at 429 nm in the solid state spectra and at 455–456 nm in solutions, see Figure S11 in Supplementary Materials. The spectra demonstrated the structural similarity of the complexes in the solvents used; however, on the other side the difference of approximately ca 30 nm between the maxima observed in the solid state and solutions showed a slight structural change associated probably with coordination of the nitrate anion to the Cu(II) atom in the solid state and/or with the coordination of the corresponding solvent in the solutions (see Figure S11 in Supplementary Materials). Mass spectra of all the complexes revealed peaks corresponding to the [Cu(L)(N–N)]^+^ complex cations, thus proving existence of these moieties in the MeOH solutions. The mass spectra of the complexes **1**–**5** are shown in Figures S12–S16 in Supplementary Materials. Molar conductivity data showed that the complexes behave as the 1:1 electrolytes in MeCN, MeOH and DMF (see Table S1 in Supplementary Materials). The detailed information describing physical and chemical characteristics of the complexes is given in the Section 3.2.

### 2.2. In Vitro Cytotoxicity of Complexes 1–5

The effect of complexes **1**–**5**, and pomiferin and cisplatin (as standards for comparative purposes) on cell viability was tested against a panel of eight human cancer cell lines (MCF-7, HOS, A549, PC-3, A2780, A2780R, Caco-2 and THP-1), and healthy human hepatocytes (Hep). The determined IC_50_ values are given in Table 1. As can be seen from the table, the most significant in vitro cytotoxicity was obtained for the complexes **3** and **5** containing the phen and dppz ligands, respectively, reaching the IC_50_ values of 2.2–13.0 μM against all the human cancer cell lines. On the other side, the complexes **1**, **2** and **4**, containing the bpy, dimebpy, and bphen ligands, respectively, revealed just moderate cytotoxicity (IC_50_ ≈ 20 μM) as for the MCF-7, HOS (except for **4**), A549 (except for **4**), PC-3 cell lines, and significant activity (IC_50_ ≈ 5–14 μM) in the case of the A2780, A2780R and Caco-2 cells as compared to cisplatin. Regarding the A2780 and A2078R cell lines, the cytotoxicity of **1**–**5** and pomiferin is comparable, whilst significantly higher as compared to cisplatin. The study of toxicity of **1**–**5** against healthy human hepatocytes showed that the IC_50_ values could not be determined within the tested concentration range of 0.1–100 μM. This fact points out the good selectivity of the complexes. As for cytotoxicity, very close and promising findings were observed for structurally similar copper(II) complexes containing osajin, a next naturally-occurred prenylated flavonoid [39,40]. The *N*,*N*′-donor ligands bipy and phen were previously [41] tested for their cytotoxicities at A2780 cell line with 24 h incubation showing cytotoxicity with IC_50_ > 100 µM.

### 2.3. Cell Cycle Modifications

**Figure 2:**
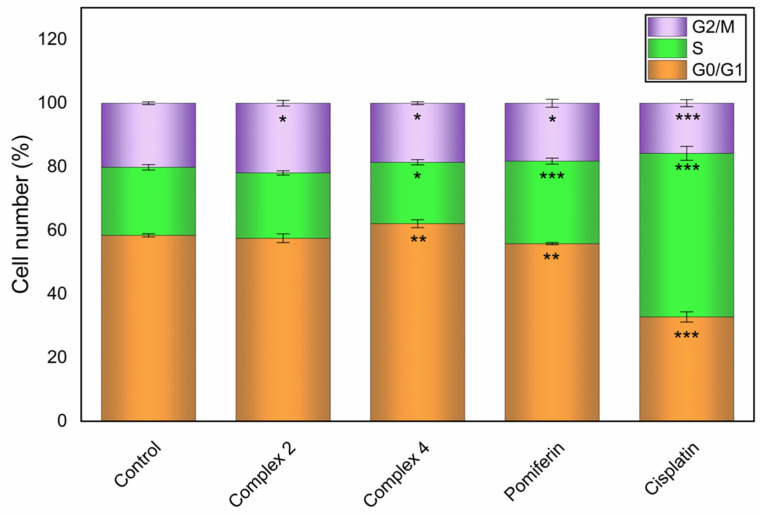
Cell cycle modifications of the A2780 cells induced by complexes **2** and **4**, pomiferin and cisplatin applied at 15 µM after 24 h of incubation. The significant change in the number of cells in a specific phase of the cell cycle caused by the respective complex as compared to the untreated control is symbolized by * at *p* ≤ 0.05 level, ** at *p* ≤ 0.001 level and *** at *p* ≤ 0.0001 level.

Aiming to understand more precisely the cellular effects of copper(II) complexes, the ability to modify the cell cycle of A2780 cancer cells of complexes **2** and **4**, which were found highly cytotoxic against this particular cell line, was studied. The biological effects of complexes **2** and **4** were compared to the effects of free ligand pomiferin and reference metallotherapeutic drug cisplatin while using the equitoxic effective concentrations of 15 µM with the incubation time of 24 h. The results expressed as a relative portion of living cells in the respective phase of a cell cycle (average values ± SD of three determinations) are shown in Figure 2. As expected, cisplatin caused a significant accumulation of cells in the S phase of the cell cycle, when compared to untreated control (51.4 ± 2.2% compared to 21.4 ± 0.8%). The observed effect of free ligand pomiferin was similar, but not as profound, as it caused an increase in cells in the S phase to 26 ± 0.9%. On the other hand, for the selected complexes **2** and **4**, the cell cycle analysis indicated a completely different mechanism of action. While the treatment with complex **4** lead to a rise in the number of cells in G0/G1 phase to 62.1 ± 1.2% vs. 58.4 ± 0.5% in control sample, complex **2** caused a slight accumulation of cells in G2/M phase 21.9 ± 0.8% vs. 20.1 ± 0.4% in control sample.

### 2.4. Induction of Cell Death and Related Processes

**Figure 3:**
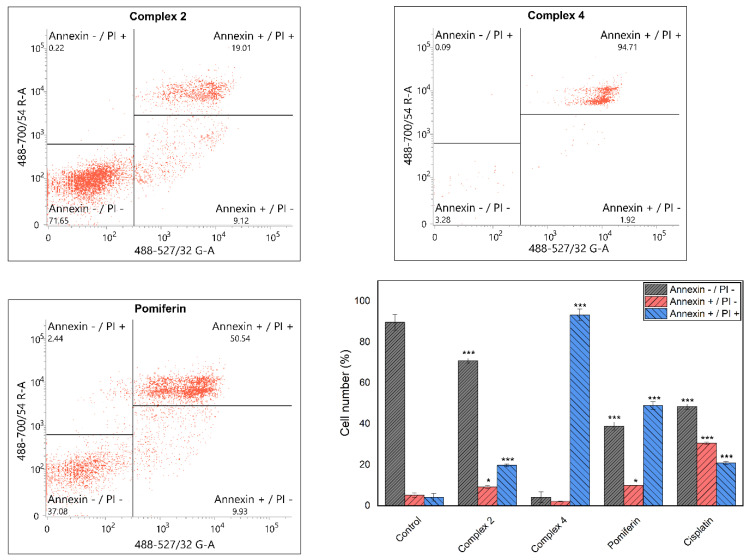
Induction of different types of cell death caused by the complexes **2** and **4**, pomiferin and cisplatin at the concentration of 15 μM after 24 h incubation. The significant change in the number of cells in a specific phase of the cell cycle caused by the respective complex as compared to the untreated control is symbolized by * at *p* ≤ 0.05 level, and *** at *p* ≤ 0.0001 level.

The induction of cell death was studied by means of the Annexin V/propidium iodide (PI) method using the flow-cytometry. This method is able to distinguish between the viable cells (Annexin V negative (−)/PI negative (−)), early apoptotic cells (Annexin V positive (+)/PI negative (−)), and late apoptotic cells/secondary necrotic cells (Annexin V +/PI +), and necrotic cells (Annexin V −/PI +) (see Figure 3 showing the flow cytometry results from one out of three independent experiments). The 24 h incubation of A2780 human cancer cells with the equitoxic concentration of complex **2** and complex **4**, both applied at 15 µM, led to completely different cellular effects, while in the case of complex **2**, the numbers of both early and late apoptotic cells increased moderately (9.2 ± 0.8% of Annexin V +/PI − cells, and 19.8 ± 0.7% of Annexin V +/PI + cells) in the case of complex **4**, almost all viable cells and early apoptotic cells were diminished and only late apoptotic cells remained (93.3 ± 2.9% of Annexin V +/PI + cells). The effect of pomiferin was found to stand between the above-mentioned two copper(II) complexes (10.0 ± 0.1% of Annexin V +/PI − cells, and 49.0 ± 1.9% of Annexin V +/PI + cells). The reference drug cisplatin, applied at the 15 µM concentration, showed the comparable induction of cell death in A2780 cells as pomiferin with a slightly higher portion of early apoptotic cells (41.5 ± 0.8% of cells), while the late apoptotic/secondary necrotic cells were less abundant (23.7 ± 2.0% of cells).

**Figure 4:**
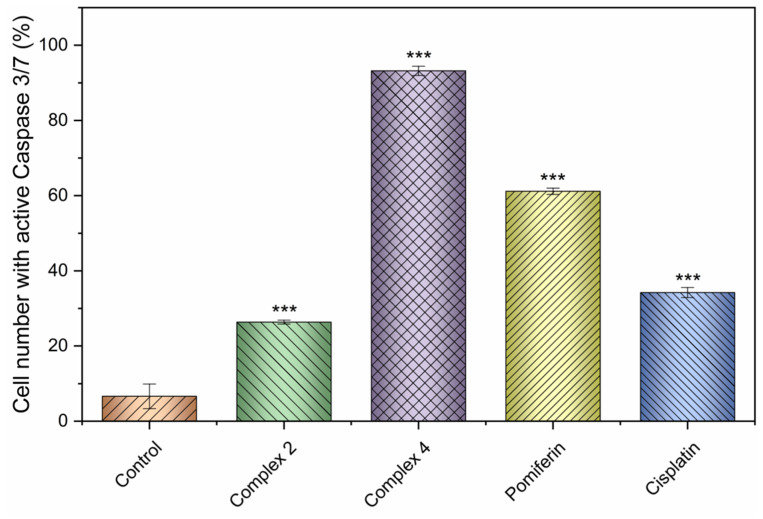
Activation of caspases 3/7 by the complexes **2** and **4**, pomiferin and cisplatin applied to the A2780 cells at 15 µM after 24 h incubation. The significant change in the number of cells with active Caspase 3/7 caused by the respective complex as compared to the untreated control is symbolized by *** at *p* ≤ 0.0001 level.

Furthermore, to the cell death assessment, we strived also to evaluate the ability of the selected complexes **2** and **4** to activate the executioner caspases 3/7. This step in the cellular metabolism is connected with the destruction of intracellular cytoskeleton and other structural proteins of the cell in the process of apoptosis. The results, presented in Figure 4, as the relative portion of A2780 cells showing the presence of activated forms of caspases 3/7 determined by the flow cytometry, revealed a good correlation between the Annexin V/PI results indicating that complex **4**, involving the bulkier 4,7-diphenyl-1,10-phenanthroline in its structure, represents the favourable structural modification, which is able to significantly increase the antiproliferative effects over the free pomiferin and reference drug cisplatin.

**Figure 5:**
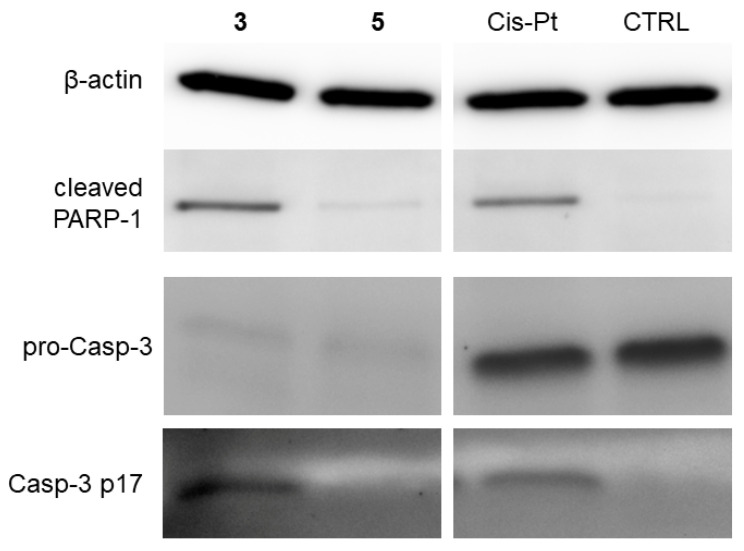
The levels of apoptosis-related proteins. THP-1 cells were treated by complexes **3** and **5** at the concentration of 4 µM and by cisplatin (Cis-Pt) at concentration of 10 µM for 24 h. Then, the amount of proteins was detected by Western blot and immunodetection. Present immunoblots show the results of pooled four independent reactions. CTRL = the cells treated only by 0.1% DMF (control cells).

In addition to the evaluation of cell death induction performed by the flow cytometric methods on A2780 cancer cells, we decided also to compare the molecular effect of two differently cytotoxic complexes **3** and **5** at the THP-1 cell line regarding the mechanisms involved in the intracellular activation of executioner caspase 3 (Casp-3) using the Western blotting. The overall results, presented in Figure 5, showed that contrary to cisplatin, used as a standard in 10 µM concentration, both of the copper(II) complexes dramatically decreased the levels of pro-caspase 3 (pro-Casp-3), an inactive precursor of Casp-3. On the other hand, the proteolytically activated Casp-3 was detected after the incubation of cells with complex **3** and cisplatin. The activity of Casp-3 was verified by the presence of cleaved poly-[ADP-ribose]-polymerase 1 (PARP-1), a substrate of Casp-3. On the other hand, only minor activity of Casp-3 was observed after application of **5**. These results indicate that complex **3** influences the molecular processes leading to the activation of Casp-3 in similar fashion as a control drug cisplatin, while complex **5** reveals a different mechanism of action.

### 2.5. Inhibition of NF-κB/AP-1 Activity

**Figure 6:**
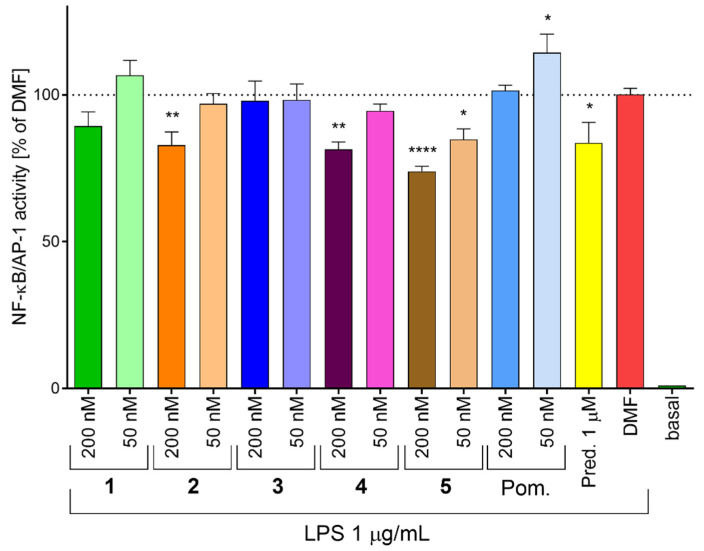
The effect of complexes **1**–**5** on the activity of transcription factors NF-κB and AP-1. THP-1-XBlue^TM^-MD2-CD14 cells were pre-treated by the complexes, pomiferin (Pom.) or prednisone (Pred.) dissolved in 0.1% DMF at indicated concentrations for 1 h. Then, lipopolysaccharide (LPS) was added to trigger the NF-κB/AP-1 activation. The activity of NF-κB/AP-1 was determined using QuantiBlue™ assay 24 h after LPS stimulation. Graph represents mean values of three determinations ± SEM. The dotted line shows the value of 0.1% DMF treated group. * indicates statistical significance (*p* ≤ 0.05) to 0.1% DMF treated group; ** indicates statistical significance (*p* ≤ 0.01) to 0.1% DMF treated group; **** indicates statistical significance (*p* ≤ 0.0001) to 0.1% DMF treated group.

Transcription factors NF-κB (nuclear factor κB) and AP-1 (activator protein 1) belong to key players in the inflammatory response [42,43,44], and thus, they represent a promising target of anti-inflammatory therapy. The complexes **1**, **2**, **4** and **5** were able to inhibit LPS-stimulated NF-κB/AP-1 activation (Figure 6), but only **2**, **4** and **5** were statistically significant. Moreover, complex **5** was active even in the concentration of 50 nM. Pomiferin itself showed any effect at the concentration of 200 nM. Interestingly, at 50 nM it slightly increased the NF-κB/AP-1 activity, although it inhibited NF-κB activation by the inhibition of IκB degradation in higher concentration (1.25 µM) [27]. These results indicate that in general, the formation of copper(II) complexes with pomiferin and *N*-donor ligands enhanced the inhibitory effect against LPS-stimulated NF-κB/AP-1 activation. Our observations suggest that this activity relates to the type of the corresponding *N*-donor ligand within the [Cu(*N–N*)]^+^ moiety, when the complexes containing bpy (complex **1**), dimebpy (complex **2**) and bphen (complex **4**) ligands had minor effect, phen (complex **3**) had no influence on the activity, and the highest activity was observed for the complex **5** involving the dppz ligand).

### 2.6. Evaluation of NF-κB Translocation

**Figure 7:**
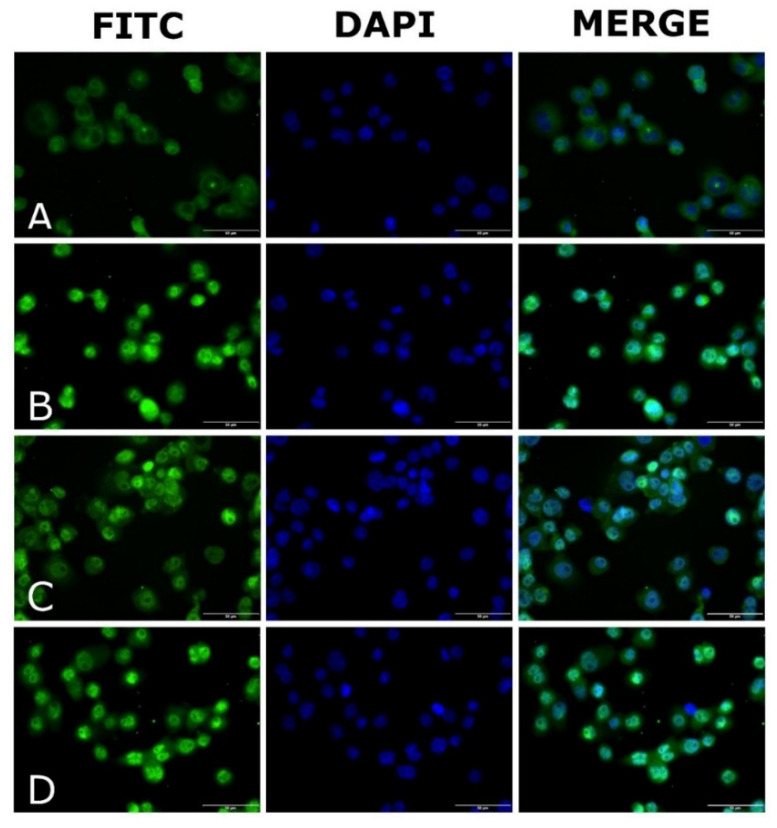
The activity of complex **5** and prednisone on NF-κB translocation. THP-1 cells differentiated into macrophages were pre-treated with **5** (200 nM) (**C**), prednisone (200 nM) (**D**) or DMF (0.1% *v/v*) (**A** = negative control, **B** = positive control) for 1 h. LPS was used for activation of NF-κB migration (**B**–**D**). The NF-κB translocation was observed by fluorescent microscope after 1 h of stimulation with LPS, the intensity of fluorescence reflects the measure of NF-κB translocation. FITC = NF-κB staining (green); DAPI = nucleus DNA staining (blue).

To elucidate the potential mechanism associated with the inhibition of NF-κB activity of complex **5**, the effect of **5** on NF-κB nuclear migration was evaluated by fluorescence microscopy (Figure 7). LPS stimulation of differentiated THP-1 macrophages led to NF-κB translocation into the nucleus. In the case of the cells treated with **5**, the NF-κB levels in the nucleus decreased. The observed effect of **5** correlates well with the above-described inhibition of NF-κB activity. These results indicate that the NF-κB pathway could be a cellular target for the tested complexes **1**–**5**.

### 2.7. Evaluation of TNF-α Secretion

**Figure 8:**
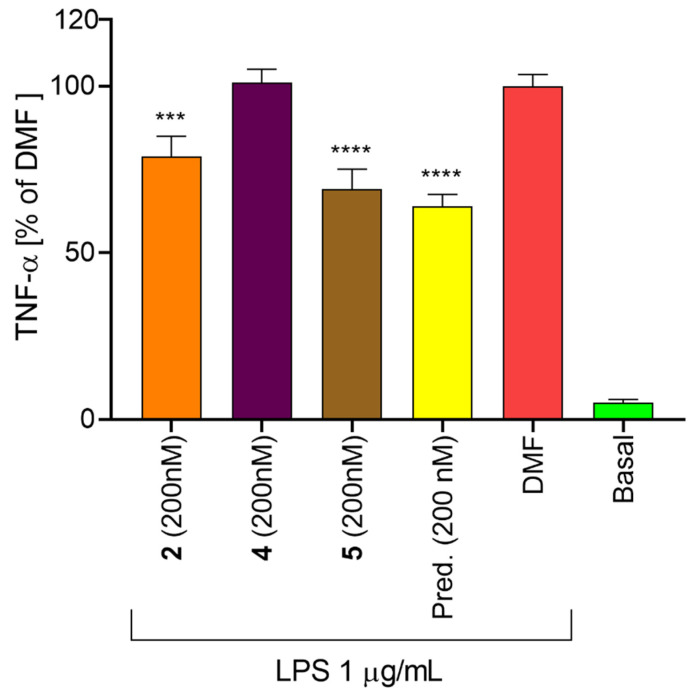
The effect of complexes **2**, **4** and **5** on the secretion of TNF-α. THP-1 cells were differentiated to macrophages by PMA. Such cells were pre-treated by complexes **2, 4,** and **5** (200 nM) or prednisone (200 nM) for 1 h, then LPS (1 µg/mL) was added to trigger the inflammatory signalization. Cultivation medium was collected 24 h after LPS stimulation and the amount of TNF-α was determined by ELISA technique. Graph represents mean ± SEM. *** indicates statistical significance (*p* ≤ 0.001) to 0.1% DMF-treated group. **** indicates statistical significance (*p* ≤ 0.0001) to 0.1% DMF-treated group.

To verify whether the copper(II) complexes possess the ability to inhibit the NF-κB/AP-1 transcription activity, the secretion of the pro-inflammatory cytokine TNF-α, which is under their transcription control [45], was evaluated after incubation of THP-1 monocytes with the representative complexes **2**, **4** and **5** (Figure 8). These complexes were chosen because of their significant effect on the NF-κB/AP-1 activity. Surprisingly, only **2** and **5** decreased its production. It can be hypothesized that complex **4**, which reduced the NF-κB/AP-1 activity, but did not affect the TNF-α synthesis, prolonged the activity of the mentioned transcription factors or the half-life of mRNA. This effect, for example, was observed in the case of natural stilbenoid thunalbene [46]. Moreover, one of our previously reported quinolinone Cu(II) complexes, containing the bphen co-ligand (similarly as complex **4** in this study), was able to increase the secretion of both TNF-α and IL-1β [14].

### 2.8. Evaluation of Apoptosis and Inflammation-Related Signalling Pathways

**Figure 9:**
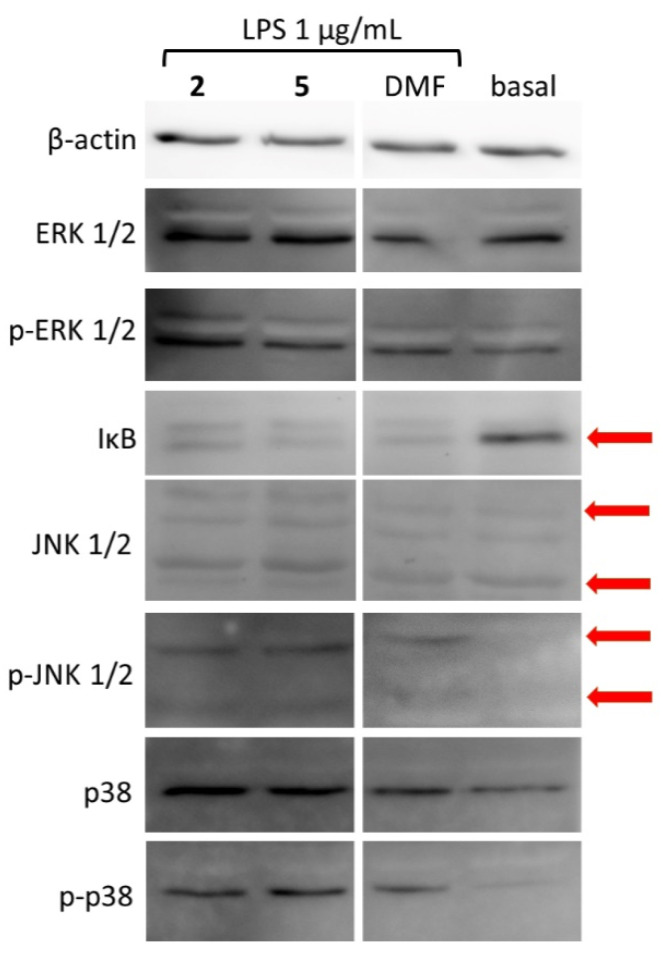
The effect of complexes **2** and **5** on the activation of MAPKs. THP-1 cells were differentiated to macrophages by PMA. The cells were pre-treated by complexes **2** and **5** (200 nM) for 1 h, then LPS (1 µg/mL) was added to trigger the inflammatory response. Cells were collected 30 min after LPS stimulation. The amount of proteins was detected by Western blot and immunodetection. Present immunoblots show the results of pooled four independent reactions. Arrows indicate the correct position of particular proteins.

The transduction of the signal from the membrane receptors and sensing of extracellular stressors to the nucleus is mediated by the system of protein kinases. The activity of the transcription factor NF-κB is regulated by its inhibitor IκB. Following the proper stimulation, this inhibitor is degraded and NF-κB is released and further translocated into the nucleus [42]. The transcription factor AP-1 is regulated by the net of up-stream mitogen-activated protein kinases (MAPKs) [44,47]. The most potent anti-NF-κB/AP-1 complexes **2** and **5** were selected for the analysis of IκB and MAPK signalling pathways to elucidate another level of mechanism of action and/or cellular targets (Figure 9). Interestingly, both the copper(II) complexes did not affect the intracellular signalization. Because complexes **2** and **5** were able to inhibit the NF-κB/AP-1 transcription activity and **4** blocked the nuclear translocation, it can be concluded that the mode of action (MoA) of these complexes lies in the modulation of cellular trafficking of transcription factors. It is worth to mention another possible MoA, such as the inhibition of binding of **2** and **5** to DNA, as was reported for Cu(II)-flavonoid complexes (see the review of Selvaraj et al., 2014 [22]), as well as for diimine co-ligands [48]; see also Section 2.11 of this article. However, further detailed analyses, involving e.g., longer incubation times and further apoptosis/inflammation signalling proteins, should be done to confirm this hypothesis.

### 2.9. Intracellular Production of Reactive Oxygen Species (ROS)

**Figure 10:**
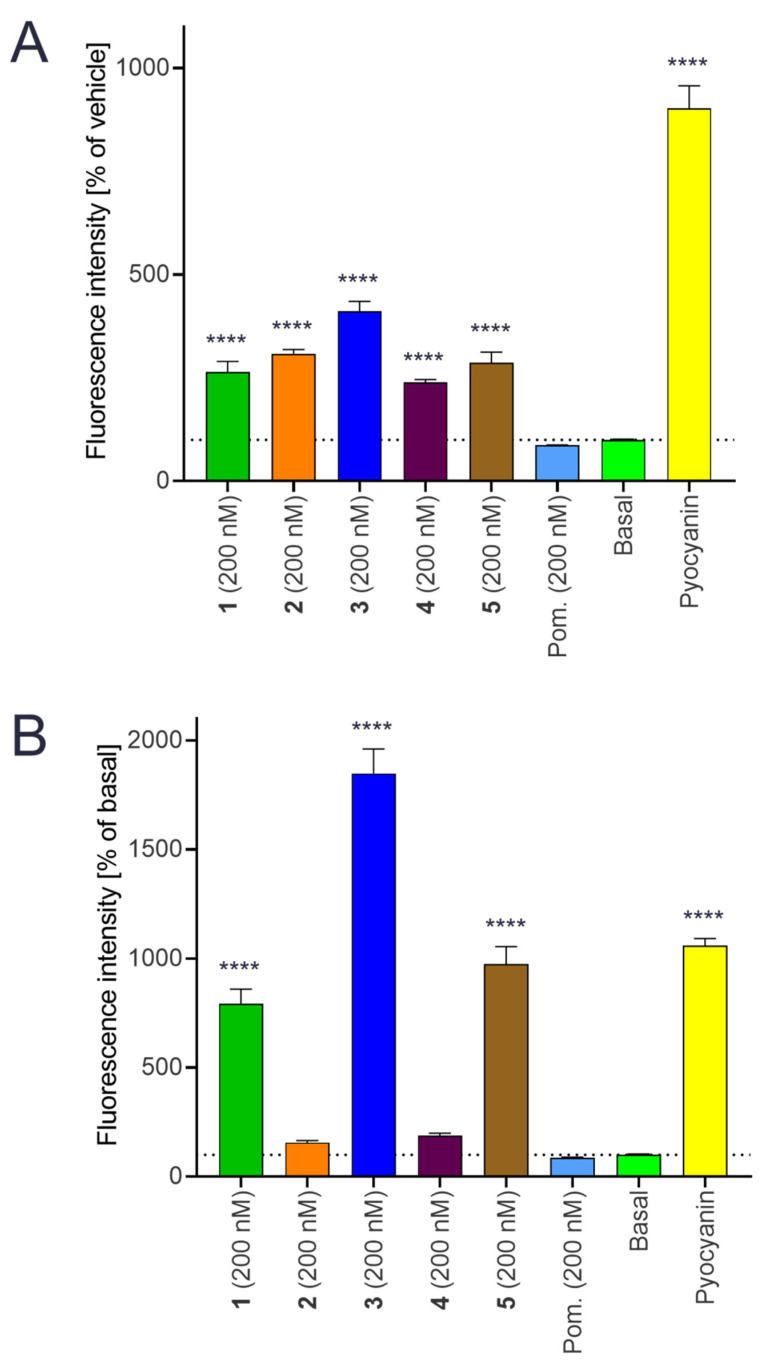
Effect of the complexes **1**–**5** on the ROS production in THP-1 cell line. The cells were treated with the complexes **1–5** (200 nM), pomiferin (Pom.; 200 nM), Pyocyanin (100 µM) or only with vehicle (basal) for 1.5 h (**A**) or 23.5 h (**B**). Then DCF-DA probe was loaded to cells and after another 30 min, the fluorescence was measured. Graph represents mean ± SEM. Dotted line show the value of basal group. **** indicates statistical significance (*p* ≤ 0.0001) to basal group.

The antioxidant and pro-oxidant potential [21,49] of flavonoids is well established in the literature. The coordination of flavonoid residue into the copper(II) complex can result either in enhancement of the antioxidant properties of the resulting complex [50] or formation of complexes showing pro-oxidant properties [51]. The pro-oxidant action of copper(II) complexes can be associated with the direct participation of the complex in Fenton-like reaction connected with the redox-cycling of the oxidation state of the central ion and/or the interaction of copper(II) complexes with native antioxidant systems, such as glutathione, thus changing the total antioxidant capacity of the system, e.g., cell, and promoting the formation of oxidative stress. To assess the ability of title copper(II) complexes to involve in the formation of the oxidative stress or to enhance it in the THP-1 cells, we performed the tests for the detection of intracellular ROS levels. All the copper(II) complexes **1**–**5** significantly increased the ROS production in cells in the short term (1.5 h) experiment (Figure 10A). In the long-term (23.5 h) period, complex **3** demonstrated the highest pro-oxidant potential of all the complexes, significantly overcoming the pro-oxidant effect of pyocyanin, a known radical-forming agent (Figure 10B). This strong pro-oxidative effect of **3** could explain, at least partially, its inability to inhibit the transcription activity of NF-κB/AP-1. It is known that NF-κB is redox sensitive and could be activated during oxidative stress [52,53] Complexes **1** and **5** elevated the ROS levels similarly as pyocyanin after 24 h incubation with THP-1 cells. The complexes **2** and **4** increased the total ROS levels by 55%, and 87%, respectively, in a 1.5 h experiment, while after 23.5 h of incubation their pro-oxidant effect almost diminished. Pomiferin itself did not increase the amount of ROS in both experiments. Similar behaviour was also observed previously for heteroleptic copper(II) complexes containing 1,4-naphthoquinone derivative lawson [54].

### 2.10. Mitochondrial Membrane Potential Modifications

**Figure 11:**
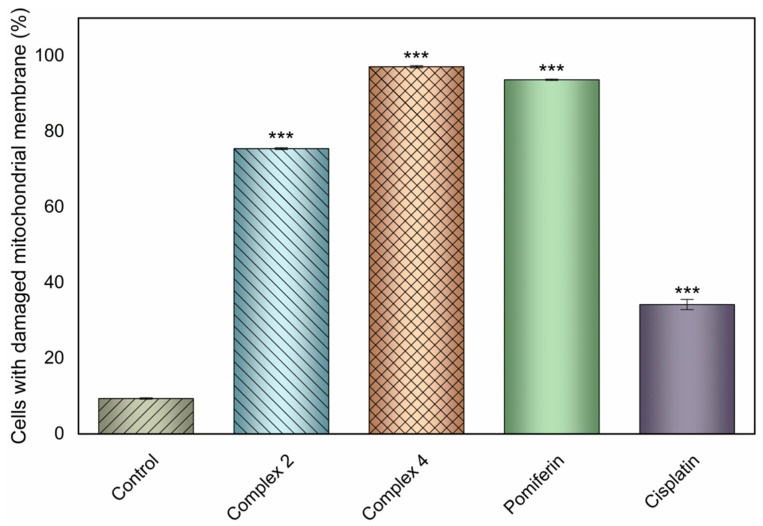
Mitochondrial membrane potential modifications induced by complexes **2** and **4**, pomiferin and cisplatin applied to the A2780 cells at 15 µM after 24 h incubation. The significant change in the number of cells with damaged mitochondrial membrane caused by the respective complex as compared to the untreated control is symbolized by *** at *p* ≤ 0.0001 level.

One of the mechanisms associated with the triggering of apoptosis is the loss of mitochondrial function and subsequent release of cytochrome c to the intracellular space [55]. The damage inflicted to the mitochondria relates to the depolarization of its membrane. To assess the ability of the selected complexes **2** and **4**, and pomiferin to potentially be involved in this mechanism of action, we performed the flow-cytometric study to determine the ratio of A2780 cancer cells, incubated with the complexes **2** and **4**, free ligand pomiferin and a reference drug cisplatin (all at 15 µM concentration) for 24 h, with damaged mitochondrial function. The results (Figure 11) showed the strong ability of both complexes **2** and **4**, as well as pomiferin, to damage the mitochondrial function in A2780 cells, significantly overcoming the action of cisplatin applied at the same concentration level.

### 2.11. Nuclease Mimicking Activity

**Figure 12:**
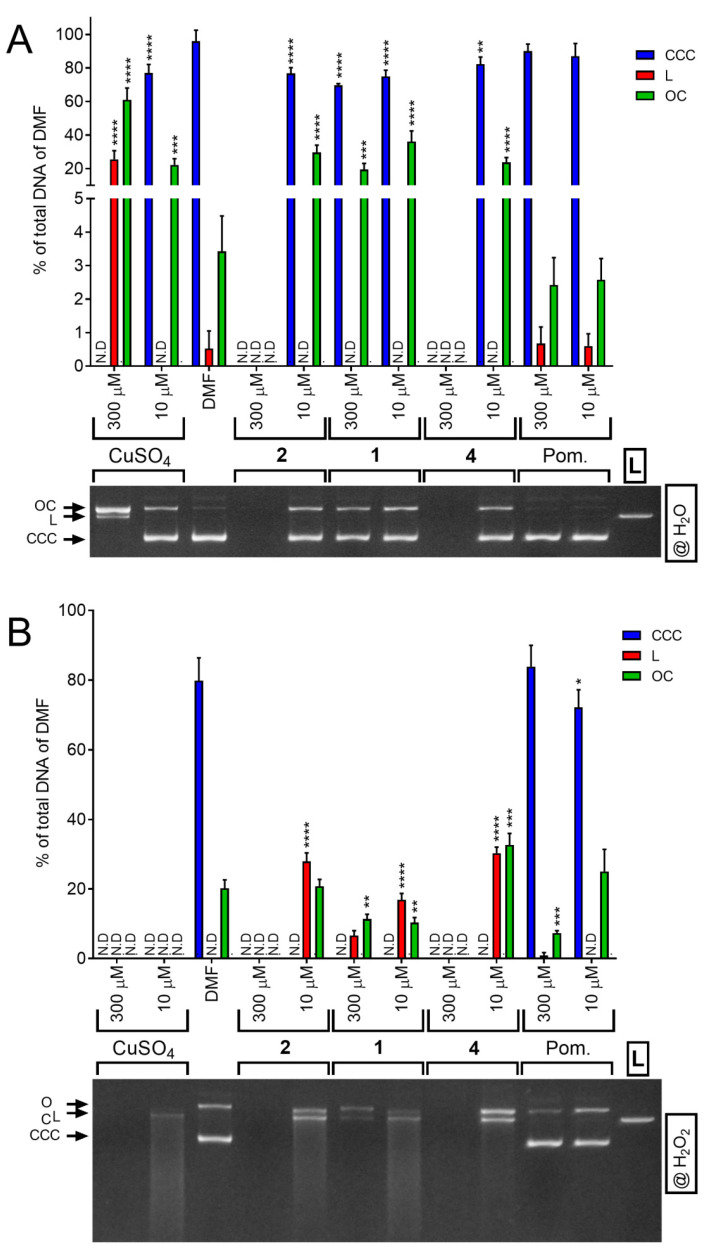
Nuclease-like effect of the tested compounds. Supercoiled plasmid DNA (CCC) was incubated with complexes **1**, **2**, and **4**, CuSO_4_·5H_2_O, pomiferin (Pom.) or pure solvent (10% DMF in water) in different concentrations at 37 °C for 1 h in water (**A**) or in 0.66 mM H_2_O_2_ (**B**). After incubation, the amount of created open circle (OC) and linear (L) form of plasmid was evaluated by densitometry analysis. Graphs show means ± SEM of four independent experiments, electrophoretograms represent the results of agarose electrophoresis. * indicates statistical difference to 10% DMF group (*p* ≤ 0.05); ** indicates statistical difference to 10% DMF group (*p* ≤ 0.01); *** indicates statistical difference to 10% DMF group (*p* ≤ 0.001); **** indicates statistical difference to DMF group (*p* ≤ 0.0001). L control linearized plasmid pUC19. N. D. = not detected. CuSO_4_ stands for water solution of copper(II) sulphate pentahydrate.

Copper complexes of phen, bpy, dppz and their derivatives are known to bind on/into DNA and possess nuclease activity [14,48,56]. DNA binding activity was also described for several flavonoid complexes (see the review of Selvaraj et al. (2014) [22]). These findings motivated us to determine the ability of selected copper(II) complexes **1**, **2**, and **4** to act as chemical nucleases using the model circular plasmid pUC19 as a primary substrate. The complexes (applied at two concentration levels, i.e., 10 µM and 300 µM) were incubated with pUC19 plasmid DNA either in a water containing media (Figure 12A) or in a solution containing hydrogen peroxide (Figure 12B) as oxidant.

In the water-containing medium, i.e., 10% DMF solution, the complexes **1**, **2** and **4** proved to be able to nick ca. 30% of the plasmid DNA and produce OC-form of the plasmid even at 10 µM concentration. At 300 µM concentration, only complexes **2** and **4** were able to cleave the DNA completely to small fragments (which could not be detected in the electrophoretograms). On the other hand, pomiferin behaved as most organic molecules and did not affect the supercoiled CCC-form of the plasmid DNA. By adding 0.66 mM H_2_O_2_ into the reaction mixtures, the ability to cleave the plasmid DNA up to smaller fragments (which were visualized as a smear in the electrophoretograms) was boosted significantly, even though the effectivity between the copper(II) complexes followed the same order, i.e., **2** ≈ **4** > **1**, and the most effective complexes **2** and **4** were comparable with the effectivity of copper sulphate pentahydrate used as a model Fenton-like reaction reagent. Pomiferin proved its antioxidant properties and lowered the portion of OC-form formed after the addition of hydrogen peroxide into the reaction significantly by ca. 20%.

The order of nuclease-like activity follows the similar structural principles as in a previous study, where copper(II) Schiff-base complexes with dimebpy showed higher effect than bpy [56].

**Figure 13:**
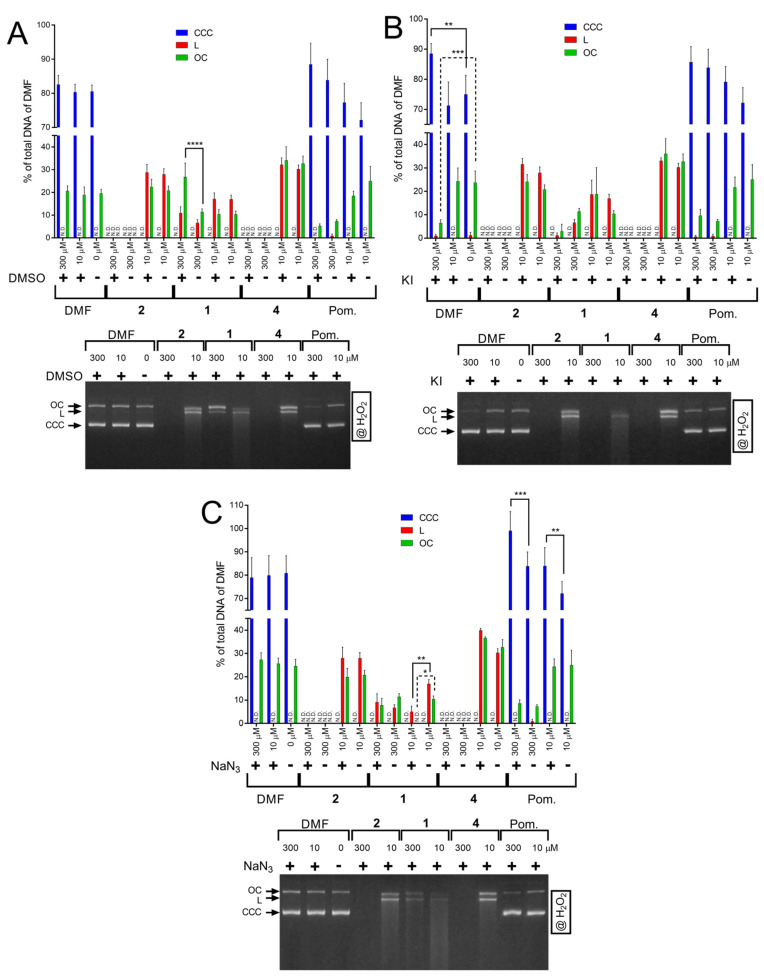
Nuclease-like effect of the tested compounds in the presence of OH^•^ scavengers DMSO (**A**), O_2_^•-^ scavengers KI (**B**), and ^1^O_2_ scavengers NaN_3_ (**C**). Supercoiled plasmid DNA (CCC) was incubated with complexes **1**, **2**, and **4**, pomiferin (Pom.) in different concentrations or pure solvent (DMF) for 1 h at 37 °C in the 0.66 mM H_2_O_2_. Scavengers were applied together with tested complexes in equimolar concentration. After incubation, the amount of created open circle (OC) and linear (L) forms of plasmid was evaluated by densitometric analysis. Graphs indicate means ± SEM of four independent experiments, electrophoretograms represent representative results of agarose electrophoresis. * indicates statistical difference between indicated groups (*p* ≤ 0.05); ** indicates statistical difference between indicated groups (*p* ≤ 0.01); *** indicates statistical difference between indicated groups (*p* ≤ 0.001); **** indicates statistical difference between indicated groups (*p* ≤ 0.0001). N. D. not detected.

As one of the main mechanisms of DNA cleavage by copper(II) complexes involves the formation of reactive oxygen species, like hydroxyl, superoxide and/or singlet oxygen, we performed the experiments involving the addition of H_2_O_2_ with the addition of different ROS scavengers, i.e., KI as a superoxide (O_2_^•-^) scavenger, NaN_3_ as a singlet oxygen (^1^O_2_) scavenger and DMSO as a hydroxyl radical (OH^•^) scavenger [57,58] (Figure 13). Interestingly, only the DNA cleavage mediated by complex **1** in the 300 µM concentration was decreased by DMSO, while the ability to cleave the DNA effectively was not affected by the addition of the ROS scavengers in the case of the most active complexes **2** and **4**. In conclusion, the DNA cleavage experiments proved the ability of all tested copper(II) complexes to interact with plasmid DNA in vitro. The results also indicated that the mechanism of DNA cleavage by the tested complexes might be rather hydrolytic than oxidative. This hypothesis can also be supported by the fact that these complexes are able to damage DNA without the presence of an oxidative agent, such as hydrogen peroxide.

## 3. Materials and Methods

### 3.1. General Methods

All reagents and solvents were received from Sigma-Aldrich or Acros and used as received without further purification, except for pomiferin, which was obtained as a generous gift from Assoc. prof. Milan Žemlička from University of Veterinary and Pharmaceutical Sciences Brno, Faculty of Pharmacy, Brno, Czech Republic. The CHN elemental analysis was performed using a CHN analyzer Flash Smart (Thermo Scientific) (Waltham, MA, USA). Infrared (IR) spectra were measured on an iS5 FT–IR spectrometer (Thermo Nicolet) (Waltham, MA, USA) using an ATR technique in the range of 400–4000 cm^−1^. Mass spectra (MS) were obtained on a Bruker amaZon mass spectrometer using ESI+ technique (Bruker amaZon SL, Bruker (Bremen, Germany)). Conductivity experiments were conducted using a Cond340i/FET apparatus (WTW, Weilheim, Germany) with 1.0 × 10^−3^ M solutions of complexes in *N,N*-dimethylformamide (DMF), acetonitrile (MeCN) and methanol (MeOH) using the Cond340i/FET conductometer (WTW). TG/DSC thermogravimetric analyses of the representative complexes **4** and **5** were carried out on an STA 449 F1 Jupiter^®^ apparatus (Netzsch, Selb, Germany) in the temperature range of 20–700 °C.

### 3.2. Preparation and Characterization of Complexes 1–5

All the complexes were synthesized by a general procedure described in detail in the patent no. CZ 308 426 B6 granted by the Industrial Property Office of the Czech Republic, and it is also a part of international patent application no. PCT/CZ2020/050050, published under no. WO/2021/018324. In brief, 0.25 mmol of 1,10-phenanthroline or 2,2′-bipyridine (or their derivatives) was dissolved in 5 mL of methanol and mixed with a solution of 0.25 mmol of Cu(NO_3_)_2_·3H_2_O dissolved in 5 mL of methanol. The reaction mixture was stirred at laboratory temperature for 15 min. Consequently, 0.25 mmol of pomiferin together with 0.34 mmol of triethylamine dissolved in 10 mL of methanol (MeOH) was added to the reaction mixture drop-wise while stirring. The reaction mixture was stirred under reflux at 65 °C for 60 min. The resulting solution was filtered, and the filtrate was left to stand at laboratory temperature for *ca* one week, when brown to green-brown powder products start to form. The solid product was separated by filtration and washed by cold methanol (2 × 5 mL) and diethyl ether (2 × 5 mL), and then dried over solid KOH in a vacuum desiccator (Fisher Scientific, Pardubice, Czech Republic).

Complex **1**: (3-(3,4-dihydroxyphenyl)-8,8-dimethyl-6-(3-methylbut-2-en-1-yl)-4H,8H-benzo[1,2-b:3,4-b′]dipyran-4-one-5-olato-κ^2^O4:O5)(2,2′-bipyridine–κ^2^N:N)copper(II) nitrate methanol disolvate; [Cu(L)(bpy)]NO_3_·2MeOH. Yield: 69%, Content of C, H, N for C_37_H_39_N_3_O_11_Cu (Mr = 765.26). Calcd.: C, 58.07; H, 5.14; N, 5.49%. Found: C, 57.98; H, 5.13; N, 5.47%. ESI+MS (methanol, m/z): 638.22 (calcd. 638.15) [Cu(L)(bpy)]^+^, 219.15 (calcd. 219.00) [Cu^I^(bpy)]^+^. IR (νATR/cm^−1^): 3208m, 3112m, 2965m, 2915m, 1627s, 1527s, 1443m, 1373m, 1297s, 1279s, 1237m, 1215m, 1184m, 1152m, 1116m, 1026s, 887w, 768m, 729w, where the letter m, s and w means the intensity; m = medium, s = strong, w = weak.

Complex **2**: (3-(3,4-dihydroxyphenyl)-8,8-dimethyl-6-(3-methylbut-2-en-1-yl)-4H,8H-benzo[1,2-b:3,4-b′]dipyran-4-one-5-olato-κ^2^O4:O5)(4,4′-dimethyl-2,2′-bipyridine–κ^2^N:N)copper(II) nitrate dihydrate; [Cu(L)(dimebpy)]NO_3_·2H_2_O. Yield: 72%, Content of C, H, N for C_37_H_39_N_3_O_11_Cu (Mr = 765.26). Calcd.: C, 58.07; H, 5.14; N, 5.49%. Found: C, 58.33; H, 5.04; N, 5.30%. ESI+MS (methanol, *m*/*z*): 666.28 (calcd. 666.18) [Cu(L)(4,4′-dimebpy)]^+^, 247.11 (calcd. 247.03) [Cu^I^(4,4′-dimebpy)]^+^. IR (νATR/cm^−1^): 3224m, 3048m, 2973m, 2923m, 1629s, 1533s, 1478m, 1413m, 1377m, 1298m, 1255s, 1214m, 1187m, 1156m, 1117m, 1048w, 1021m, 997m, 820m, 726m.

Complex **3**: (3-(3,4-dihydroxyphenyl)-8,8-dimethyl-6-(3-methylbut-2-en-1-yl)-4H,8H-benzo[1,2-b:3,4-b′]dipyran-4-one-5-olato-κ^2^O4:O5)(1,10-phenanthroline–κ^2^N:N)copper(II) nitrate methanol disolvate; [Cu(L)(phen)]NO_3_·2MeOH. Yield: 78%, Content of C, H, N for C_39_H_39_N_3_O_11_Cu (M r= 789.29). Calcd.: C, 59.35; H, 4.98; N, 5.32%. Found: C, 59.65; H, 5.06; N, 5.41%. ESI+MS (methanol, m/z): 662.28 (calcd. 662.15), [Cu(L)(phen)]^+^, 243.09 (calcd. 243.00) [Cu^I^(phen)]^+^. IR (νATR/cm^−1^): 3562w, 3297w, 3083m, 2971m, 2916m, 1633s, 1540s, 1448m, 1389s, 1322s, 1305m, 1273m, 1241m, 1217s, 1148m, 1113m, 1090m, 842m, 719m.

Complex **4**: (3-(3,4-dihydroxyphenyl)-8,8-dimethyl-6-(3-methylbut-2-en-1-yl)-4H,8H-benzo[1,2-b:3,4-b′]dipyran-4-one-5-olato-κ^2^O4:O5)(4,7-diphenyl-1,10-phenanthroline–κ^2^N:N)copper(II) nitrate methanol solvate; [Cu(L)(bphen)]NO_3_·MeOH. Yield: 69%, Content of C, H, N for C_50_H_43_N_3_O_10_Cu (Mr = 909.44). Calcd.: C, 66.03; H, 4.77; N, 4.62%. Found: C, 65.86; H, 4.81; N, 4.52%. ESI+MS (methanol, m/z): 814.39 (calcd. 814.21) [Cu(L)(bphen)]^+^. IR (νATR/cm^−1^): 3360m, 3060m, 2973m, 2916m, 1627s, 1525s, 1422m, 1298m, 1214m, 1185m, 1155w, 1116m, 1071w, 1030w, 997w, 927w, 854m, 765m, 735m, 700m.

Complex **5**: (3-(3,4-dihydroxyphenyl)-8,8-dimethyl-6-(3-methylbut-2-en-1-yl)-4H,8H-benzo[1,2-b:3,4-b′]dipyran-4-one-5-olato-κ^2^O4:O5)(dipyrido[3,2-a:2′,3′-c]phenazine–κ^2^N4:N5)copper(II) nitrate methanol solvate; [Cu(L)(dppz)]NO_3_·MeOH. Yield: 82%, Content of C, H, N for C_44_H_37_N_5_O_10_Cu (Mr = 859.34). Calcd.: C, 61.50; H, 4.34; N, 8.15%. Found: C, 61.77; H, 4.23; N, 8.28%. ESI+MS (methanol, m/z): 764.34 (calcd. 764.17) [Cu(L)(dpp)]+. IR (νATR/cm^−1^): 3077m, 2973m, 2911m, 1624s, 1584m, 1514s, 1413m, 1356m, 1302s, 1205m, 1155m, 1116m, 1078m, 1030w, 998w, 823m, 764m, 727m.

### 3.3. In Vitro Cytotoxicity

The in vitro cytotoxicity of the prepared complexes **1**–**5**, and pomiferin and cisplatin for comparative purposes, was determined by the MTT assay as described previously [39,40]. The testing was performed on the following human cancer cell lines: breast adenocarcinoma (MCF-7), osteosarcoma (HOS), lung adenocarcinoma (A549), prostate adenocarcinoma (PC-3), ovarian carcinoma (A2780), cisplatin-resistant ovarian carcinoma (A2780R) and colorectal adenocarcinoma (Caco-2) obtained from ATCC collection of cell lines and cultivated according to producer’s instructions.

The THP-1 human monocytic leukemia cell line was purchased from the European Collection of Cell Cultures (ECACC, Salisbury, UK). Cells were cultured in an RPMI 1640 medium containing stabilized 2 mM L-glutamine (Biosera, Nuaille, France) supplemented with the mixture of antibiotics [100 U/mL penicillin and 100 mg/mL streptomycin (Biosera, Nuaille, France)], and 10% foetal bovine serum (FBS) (HyClone, Marlborough, MA, USA). The cells were kept in an incubator at 37 °C in an atmosphere of air containing 5% CO_2_ and 100% humidity.

The relative cell viability of floating monocytes THP-1 were determined after 24 h incubation in a serum-free medium with compounds **1**–**5** dissolved in *N*,*N*-dimethylformamide (DMF) at increasing concentrations (20–0.125 µM) by the cell proliferation reagent WST-1 (Roche, Basel, Switzerland) according to the manufacturer’s manual, as we described previously [54]. The IC_50_ values were calculated from resultant viability curves by four-parameter logistic (4PL) analysis.

### 3.4. Cell Cycle Analysis

Human ovarian cancer cell line A2780 (Sigma, Darmstadt, Germany) were used for this study. Cells were cultivated at 37 °C under a 5% CO_2_ atmosphere in an RPMI 1640 medium (Sigma Aldrich, Darmstadt, Germany) supplemented with (the final concentrations in medium): L-glutamine (2 mM), fetal bovine serum (FBS, 10%) and PenStrep (5 U penicillin, 50 µg streptomycin/mL).

We seeded 10^4^ cells/well in 96-wells and the next day, the cells were treated with 15 µM of copper(II) complexes **2** and **4** or pomiferin, or 15 µM of cisplatin used as a reference standard. After 24 h, the cells were washed once with PBS (0.1 M, pH 7.4) and cell cycle analysis was performed according to the protocol of BD Cycletest^TM^ Plus DNA kit (Becton Dickinson, Franklin Lakes, NJ, USA). Measurements were performed using BD FACSVerse flow cytometer (Becton Dickinson, Franklin Lakes, NJ, USA) in 3 independent experiments, each done in duplicate, while at least 5 × 10^3^ events were recorded for each sample.

### 3.5. Induction of Cell Death and Related Processes

The ability of selected copper(II) complexes **2** and **4**, pomiferin and cisplatin (all applied at 15 µM concentration) to induce the cell death in A2780 cells was evaluated using 2 methods: (i) Early/late stage of apoptosis was detected using the Annexin V-FITC apoptosis detection kit (Enzo Life Sciences, Farmingdale, NY, USA); and (ii) Caspase 3/7 induction was determined by the CellEvent^TM^ Caspase-3/7 Green Flow Cytometry Assay Kit (Thermo Fisher Scientific, Waltham, MA, USA). Both methods were performed according to the manufacturers’ protocols with 1 modification in the caspase induction assay, which was the use of the CellEvent^TM^ Caspase-3/7 Green Detection Reagent only for detection of Caspase-3/7 activation. We seeded 5 × 10^3^ cells/well in 24-well plate and the next day, cells were treated with 15 µM of copper(II) complexes, pomiferin, or 15 µM of cisplatin. After 24 h, the cells were washed once with PBS (0.1 M, pH 7.4), detached with trypsin (0.25% in ethylenediaminetetraacetic acid (EDTA), Sigma-Aldrich), resuspended in 500 µL of the culture medium and divided into 2 separate tubes, the first for apoptosis assay and the second one for caspase activation analysis. After staining with appropriate dyes, samples were analyzed on a BD FACSVerse flow cytometer (Becton Dickinson, Franklin Lakes, NJ, USA) in 3 separate experiments, while at least 10^4^ events were recorded for each sample prepared in duplicate.

In addition to the above-mentioned methods, the Western blotting method was used to shed light on the mechanisms associated with caspase 3 activation in the THP-1 cells. Two and a half milliliters of floating THP-1 monocytes resuspended in a serum-free RPMI 1640 medium were seeded into 6-well plates at the concentration of 10^6^ cells/mL. Two hours later, complexes **3** and **5** were added at the concentration level of 4 µM. After 24 h incubation, the cells were collected by centrifugation (3000 g/5 min/4 °C), washed by cold PBS and resuspended in an ice-cold lysis buffer (50 mM Tris-HCl pH 7.5, 1 mM EGTA, 1 mM EDTA, 1 mM sodium orthovanadate, 50 mM sodium fluoride, 5 mM sodium pyrophosphate, 270 mM sucrose) with Roche cOmplete protease inhibitors (Roche Diagnostics, Basel, Switzerland). The cell lysates were centrifuged at 3000 g/5 min/4 °C and supernatants were mixed with a 5x Laemmli buffer [containing 250 mM Tris-HCl pH 6.8, 10% (*w/v*) SDS, 30% (*v/v*) glycerol, 5% (*v/v*) β-mercaptoethanol, 0.04% (*w/v*) bromphenol blue] and incubated at 70 °C for 5 min. To separate the proteins, 6 μg of the proteins were loaded onto a 12% polyacrylamide gel and then transferred electrophoretically to PVDF (polyvinylidene fluoride) membranes that were subsequently blocked using 5% BSA dissolved in a TBST buffer [containing 150 mM NaCl, 10 mM Tris base and 0.1% (*v/v*) Tween-20]. The membranes were incubated at 4 °C for 16 h with a primary anti-cleaved caspase 3 (p17) antibody at the dilution 1:1000 (Cell Signaling, Danvers, MA, USA; products No. 9664), and an Apoptosis WB Cocktail (Abcam, Cambridge, UK; product No. ab136812) at the dilution recommended by the provider to detect β-actin, cleaved poly-(ADP-ribose) polymerase (PARP) and pro-caspase 3. After washing in a TBST buffer, the secondary anti-rabbit IgG antibody (Sigma-Aldrich, product No. A0545) was diluted 1:2500 and applied to the membranes, which were then incubated for 1 h at the laboratory temperature (~22 °C). The amount of bound secondary antibody was determined using an ECL reagent (Bio-Rad, Hercules, CA, USA). Chemiluminescence was detected using a Syngene PXi4 chemiluminescence imaging system (Cambridge, UK).

### 3.6. Detection of the Activation of NF-κB/AP-1

The activity of transcription factors NF-κB/AP-1 was evaluated using the THP-1-XBlue^TM^-MD2-CD14 cell line [obtained from Invivogen (San Diego, CA, USA)], expressing an NF-κB/AP-1-inducible secreted embryonic alkaline phosphatase (SEAP) reporter gene, as was described previously [54,59]. The complexes **1**–**5** were applied to the cells at the concentrations of 200 nM and 50 nM, pomiferin (at 200 nM), and a reference drug prednisone at 1 μM, respectively. Lipopolysaccharide (LPS) from *E. coli* 0111:B4 (Sigma-Aldrich) dissolved in PBS was used to trigger an inflammation-like reaction. The activity of the SEAP was determined by Quanti-Blue^TM^ reagent (Invivogen, San Diego, CA, USA) according to the manufacturer’s instructions 24 h after lipopolysaccharide stimulation. The activity of NF-κB/AP-1 was determined spectrophotometrically using the FLUOstar Omega microplate reader (BMG Labtech, Ortenberg, Germany) at 655 nm and compared with that of the vehicle.

### 3.7. Immunocytochemical Analysis of NF-κB Nuclear Translocation

To differentiate the THP-1 monocytes to macrophages, phorbol myristate acetate (PMA) was used at the final concentration of 50 ng/mL. The cells were split into 8-well cell culture glass slides (SPL, Gyeonggi-do, South Korea) at a concentration of 3 × 10^5^ cells/300 µL/well. Monocytes were differentiated by PMA treatment for 24 h and then the medium was changed. Macrophages were washed with PBS, transferred into a serum-free medium and incubated for 2 h. After 1 h pre-treatment with **5** (200 nM), prednisone (200 nM) or 0.1% DMF (*v/v*) solution used as a control, NF-κB nuclear translocation was triggered by the addition of LPS (1 µg/mL). The macrophages were washed with PBS and fixed with 4% formaldehyde 1 h later. Permeabilization was performed using 0.1% *v/v* Triton X-100 (Merck). After blocking with 3% bovine serum albumin (BSA; Merck, Darmstadt, Germany) dissolved in PBS, the primary antibody anti-NF-κB p65 (Abcam, Cambridge, UK, product No. ab16502) was added at a concentration of 2.5 µg/mL and incubated at 4 °C overnight. Then a secondary anti-rabbit antibody labelled by Alexa Fluor 488^®^ (Cell Signalling, Danvers, MA, USA) product No. 4412, dilution 1:1500) was applied in dark at 4 °C for 1 h. The nuclei were stained with DAPI (Merck, Darmstadt, Germany). PBS was used for washing the wells between every individual steps 3 times. The coverslip was mounted with VectaShield mounting medium (Vector laboratories, Burlingame, CA, USA) and sealed with nail polish. The NF-κB migration activity was evaluated by fluorescent microscopy after the nail polish cured using the fluorescence microscope IX73 (Olympus, Tokyo, Japan).

### 3.8. Evaluation of TNF-α Secretion

Differentiated THP-1 macrophages (see above) were pre-treated with either 200 nM solutions of the tested complexes, or 200 nM solution of prednisone dissolved in 0.1% DMF or the vehicle (0.1% (*v/v*) DMF solution) for 1 h, as described previously [54,59]. The concentration of TNF-α was measured using a Human TNF-α ELISA Kit (Diaclone, Besançon, France), according to the manufacturer’s manual. Each experiment was conducted in triplicate.

### 3.9. Apoptosis and Inflammation-related Signalling Pathways Analysis

Macrophages derived from THP-1 cells were used to evaluate the influence of tested complexes on the signalling pathway leading to activation of transcription factors NF-κB and AP-1. To differentiate THP-1 monocytes into macrophages, the cells were stimulated with phorbol myristate acetate (PMA), as described above. The level of inhibitor of NF-κB (IĸB) and phosphorylation (activation) of mitogen-activated protein kinases (MAPKs) p38, ERK1/2, and JNK was determined in a way similar to that described previously [46]. Above mentioned proteins were detected by the following primary antibodies: anti-IĸB-α at the dilution 1:500 (Cell Signalling, Danvers, MA, USA; products No. 4814), anti-phospho-SAPK/JNK at the dilution 1:500 (Cell Signalling; products No. 4668), anti-p44/42 MAPK (ERK1/2) at the dilution 1:1000 (Cell Signalling; products No. 4695), anti-phospho-p44/42 MAPK (ERK1/2) at the dilution 1:1000 (Cell Signalling; products No. 4370), anti-p38 MAPK at the dilution 1:1000 (Cell Signalling; products No. 9212), anti-phospho-p38 MAPK antibodies at the dilution 1:1000 (Cell Signalling; products No. 4511), anti-SAPK/JNK at a dilution of 1:250 (Sigma-Aldrich, product No. SAB4200176), and anti-β-actin at a dilution of 1:5000 (Abcam, Cambridge, UK; product No. 8226). After washing, the secondary anti-mouse and anti-rabbit IgG antibodies (Sigma-Aldrich, products No. A0168 and A0545) were diluted 1:2500 and applied to the membranes. The amount of bound secondary antibody was determined using ECL reagent (Bio-Rad, Hercules, CA, USA). Chemiluminescence was detected using a Syngene PXi4 chemiluminescence imaging system (Cambridge, UK).

### 3.10. Detection of the Intracellular Production of Reactive Oxygen Species

THP-1 cells were used for the determination of the potential of tested compounds to initiate the intracellular ROS production, as was described previously with some minor modifications [54,59]. Cells in serum-free RPMI 1640 medium without phenol red were seeded into black 96-well plates (5 × 10^4^ cells/100 µL/well). After 2 h of incubation, the cells were treated with copper(II) complexes **1**–**5** at a concentration of 200 nM, and pyocyanin at a final concentration of 100 μM was added into control group to trigger production of reactive oxygen species (ROS), respectively. After the incubation at 37 °C for either 1.5 h or 23.5 h, dichlorofluorescin diacetate (DCFH_2_-DA) (5 μg/mL) was added and cells were kept under the same conditions hereafter. Thirty minutes later, fluorometric measurement of the fluorescent product DCF was conducted using a Tecan Infinite F200 microplate reader (Tecan, Männedorf, Switzerland) with λ_(ex./em.)_ = 485/520 nm.

### 3.11. Mitochondrial Membrane Potential Modifications

For mitochondrial membrane potential analysis, 5 × 10^4^ A2780 cells/well were collected in 24-well plate and treated with complexes **2** and **4,** pomiferin and cisplatin (at the final concentration of 15 µM) the next day. After 24 h, the cells were washed once with PBS (0.1 M, pH 7.4), detached with trypsin (0.25% in ethylenediaminetetraacetic acid (EDTA), Sigma-Aldrich), and resuspended in 500 µL of culture medium. Then the staining was performed using MITO-ID^®^ Membrane potential detection kit (Enzo Life Sciences, Farmingdale, NY, USA) according to the manufacturer’s protocol and samples were measured in three independent experiments, each time in duplicates on BD FACSVerse flow cytometer (Becton Dickinson, Franklin Lakes, NJ, USA), while at least 10^4^ events were recorded for each sample.

### 3.12. Nuclease-like Activity Experiments

The nuclease activity of the complexes **2**, **3**, and **5,** and pomiferin was determined using the previously published method with small modifications [60]. Three hundred nanograms of the native supercoiled pUC19 plasmid DNA (i.e., 23.1 µM of base pairs in the final volume of 20 µL of reaction mixture) was incubated for 1 h either in the presence or in the of absence of 0.66 mM H_2_O_2_, together with different concentrations of the tested compounds dissolved in 0.1% *N,N*-dimethylformamide (DMF) at the concentration levels of 0, 10 and 300 µM at 37 °C for 1 h in the dark. Immediately after that, the samples were quickly mixed with 6× gel loading buffer (containing 60 mM EDTA, 60% (*v/v*) glycerol, 0.03% (*w/v*) bromphenol blue) and subsequently loaded on a 0.8% (*w/v*) agarose gel prepared in TBE buffer (containing 89 mM Tris-borate buffer and 2 mM EDTA; Sigma-Aldrich) impregnated with 0.15 µg/mL of ethidium bromide (EtBr). The electrophoretogram was analyzed by the AlphaEaseFC version 4.0.0.34 software (Alpha Innotech, San Leandro, CA, USA) and the relative amounts of the supercoiled circular (CCC-form), single-strand nicked (OC-form) and linear (L-form) forms of plasmid DNA were evaluated. The quantification of CCC-form of plasmid DNA was corrected by a factor of 1.47 [57]. The relative amount of each plasmid form was calculated as a percentage of the total amount of DNA in the negative control, containing the native form of plasmid DNA only (i.e., containing only solvent without the compounds).

### 3.13. Isolation of pUC19 Plasmid DNA for Nuclease Experiments

The ability of metal complexes to act as chemical nucleases was evaluated using the pUC19 plasmid (2686 bp). Plasmids were isolated from the transformed bacteria *Escherichia coli* TOP10F’ by the QIAprep Spin Miniprep Kit (Qiagen, Hilden, Germany) according to the manufacturer’s instruction. The plasmids’ DNA was eluted from the column by nuclease-free ultrapure water and it was used in the DNA cleavage assays. The quantity and purity of isolated DNA was measured spectrophotometrically at 230, 260, 280 and 320 nm. The absorbance ratio A_260_/A_280_ was in the range between 1.75 and 1.85, and the absorbance ratio A_260_/A_230_ was in the range between 2.00 and 3.00, which confirmed that the DNA was free of proteins, RNA and other impurities.

### 3.14. Effect of the Antioxidants and Other Inhibitors on the Plasmid DNA Cleavage

To reveal the participation of reactive oxygen species or other mechanisms in the cleavage processes of the plasmid DNA, the reaction mixtures were supplemented with the various ROS scavengers, specifically NaN_3_, (a selective quencher of singlet oxygen [61]*),* DMSO and KI (a highly effective scavenger of hydroxyl [57,62]). All antioxidants were added to the reaction mixtures in the molar ratio of 1:1 with 300 µM and 10 µM concentrations of the complexes and these were incubated with the plasmid DNA in a similar manner as described above.

### 3.15. Statistical Evaluations

Statistical analyses were carried out using GraphPad Prism 6.01 software (San Diego, CA, USA). Outlier values were identified by ROUT algorithms (Q = 5%) and excluded from the analysis. The data were graphed as means ± SEM. Comparisons between groups were made using a one-way ANOVA test followed by a Fisher’s LSD post-hoc test. For cell cycle, apoptosis and oxidative stress analysis, we performed 3 independent experiments and the standard error of mean (±SEM) was calculated. One-way ANOVA was performed using Statistica software, version 14.0 (TIBCO, Palo Alto, CA, USA) and significant difference compared to negative control was highlighted. Any difference was considered significant at *p* ≤ 0.05.

## 4. Conclusions

A series of five new heteroleptic copper(II) pomiferin-containing complexes were prepared and thoroughly characterized. The complexes were found as effective antiproliferative agents revealing significant in vitro cytotoxicity against a panel human cancer cell lines (MCF-7, HOS, A549, PC-3, A2780, A2780R, Caco-2 and THP-1), with the IC_50_ values of 2.2–13.0 μM for the best performing complexes **3** and **5**. Moreover, the complexes showed relatively low toxicity on healthy human hepatocytes, with IC_50_ > 100 μM. In addition to that, the results following from an Annexin V/PI apoptosis assay, induction of intracellular reactive oxygen species (ROS) production, perturbation of mitochondrial membrane potential and nuclease mimicking activity revealed their ability to induce the processes leading to the destruction of intracellular molecules of life and subsequent cell death, dominantly via the initiation or progression of oxidative stress. On the other hand, the complexes showed an ability to inhibit the inflammation-related signalling pathways (NF-κB/AP-1 activity, NF-κB translocation and TNF-α secretion), thus rendering the tested complexes as effective anti-inflammatory substances. Overall, the results showed significant biological potential of the studied compounds and revealed that copper(II) complexes, involving the naturally occurring ligand pomiferin with intrinsic biological activities, deserve to be studied deeper and in greater detail in the future.

## Figures and Tables

**Table 1:** The results of in vitro cytotoxicity testing (expressed as mean IC_50_ values with standard deviations (SD) calculated out of at least three independent experiments in μM) of complexes **1**–**5** on the following human cancer cell lines: breast adenocarcinoma (MCF-7), osteosarcoma (HOS), lung adenocarcinoma (A549), prostate adenocarcinoma (PC-3), ovarian carcinoma (A2780), ovarian carcinoma resistant to cisplatin (A2780R), colorectal carcinoma (Caco-2), and THP-1, and toxicity against primary culture of human hepatocytes (Hep). The cytotoxicity of the complexes is compared to the free ligand HL (pomiferin) and platinum-containing chemotherapeutic drug cisplatin.

Comp.	MCF-7	HOS	A549	PC-3	A2780	A2780R	Caco-2	THP-1 *	Hep
**1**	23.8 ± 0.2	21.5 ± 1.4	20.8 ± 1.5	22.1 ± 1.9	12.1 ± 1.5	5.6 ± 0.3	13.7 ± 7.4	4.0 ± 1.1	>100
**2**	29.6 ± 2.0	21.1 ± 1.6	20.9 ± 0.9	>50	5.4 ± 2.4	5.3 ± 1.1	5.1 ± 1.6	7.9 ± 1.1	>100
**3**	5.9 ± 0.6	3.5 ± 0.1	3.5 ± 0.2	13.0 ± 1.4	5.8 ± 2.2	2.9 ± 1.2	4.2 ± 1.9	>10	>100
**4**	16.0 ± 1.1	2.6 ± 0.5	8.7 ± 0.8	13.0 ± 1.1	12.8 ± 3.1	6.6 ± 2.3	22.9 ± 12.9	3.3 ± 1.2	>100
**5**	3.0 ± 0.5	2.4 ± 0.9	3.1 ± 0.2	3.3 ± 0.1	2.5 ± 0.9	2.2 ± 0.5	2.6 ± 1.6	3.1 ± 1.1	>100
pomiferin	22.0 ± 0.1	20.7 ± 0.5	21.2 ± 1.4	18.5 ± 0.7	8.4 ± 2.0	4.3 ± 1.3	19.8 ± 2.4	>10	>50
cisplatin	30.8 ± 1.8	30.0 ± 5.9	>80	>80	26.3 ± 2.1	>80	>80	n.t.	>80

* incubated with the tested complexes in serum-free medium for 24 h, result calculated from six independent experiments n.t. = not tested.

## Data Availability

The data presented in this study are available online in this article and supplementary materials at www.mdpi.com/xxx/s1.

## Supplementary Materials

The following are available online at https://www.mdpi.com/article/10.3390/ijms22147626/s1. IR Spectrum of [Cu(L)(bpy)]NO_3_·2MeOH (**1**) (Figure S1). IR Spectrum of [Cu(L)(dimebpy)]NO_3_·2H_2_O (**2**) (Figure S2). IR Spectrum of [Cu(L)(phen)]NO_3_·2MeOH (**3**) (Figure S3). IR Spectrum of [Cu(L)(bphen)]NO_3_·MeOH (**4**) (Figure S4). IR Spectrum of [Cu(L)(dppz)]NO_3_·MeOH (**5**) (Figure S5). IR spectrum of pomiferin (**HL**) (Figure S6). TG/DSC curves of complexes **4** and **5** (Figures S7 and S8, respectively). Diffuse reflectance U*V/V*is spectra of complexes **1**–**5** measured in solid state (Nujol mulls) (Figure S9). U*V/V*is spectra of complexes **1**–**5** measured in DMF solutions at the 10 µM concentration (Figure S10). U*V/V*is spectra of complex **5** (Figure S11). Mass spectra of complexes **1, 2, 3, 4,** and **5** (Figures S12–S16). The values of molar conductivity of complexes **1**–**5** in different solvents (Table S1).
