# Supplementary material for: Copper(II) Complexes Containing Natural Flavonoid Pomiferin Show Considerable In Vitro Cytotoxicity and Anti-inflammatory Effects
Source: Int J Mol Sci. 2021 Jul 16;22(14):7626. doi: 10.3390/ijms22147626 (PMC8305652; doi:10.3390/ijms22147626)
Supplement: Supplementary file 1 [file ijms-22-07626-s001.zip › ijms-1291611-supplementary.pdf]

Supplementary Materials

Copper(II) complexes containing natural flavonoid pomiferin show considerable in vitro cytotoxicity and anti-inflammatory effects

Ján Vančo [1], Zdeněk Trávníček [1,*] Jan Hošek [1], Tomáš Malina[1], and Zdeněk Dvořák [2]

[1] Regional Centre of Advanced Technologies and Materials, Czech Advanced Technology and Research Institute (CATRIN), Palacký University, Šlechtitelů 27, CZ-783 71 Olomouc, Czech Republic, jan.vanco@upol.cz (J.V.); jan.hosek@upol.cz (J.H.); zdenek.travnicek@upol.cz (Z.T.)

[2] Department of Cell Biology and Genetics, Faculty of Science, Palacký University, Šlechtitelů 27, CZ-783 71 Olomouc, Czech Republic, zdenek.dvorak@upol.cz (Z.D.)

* Correspondence: zdenek.travnicek@upol.cz (Z.T.)

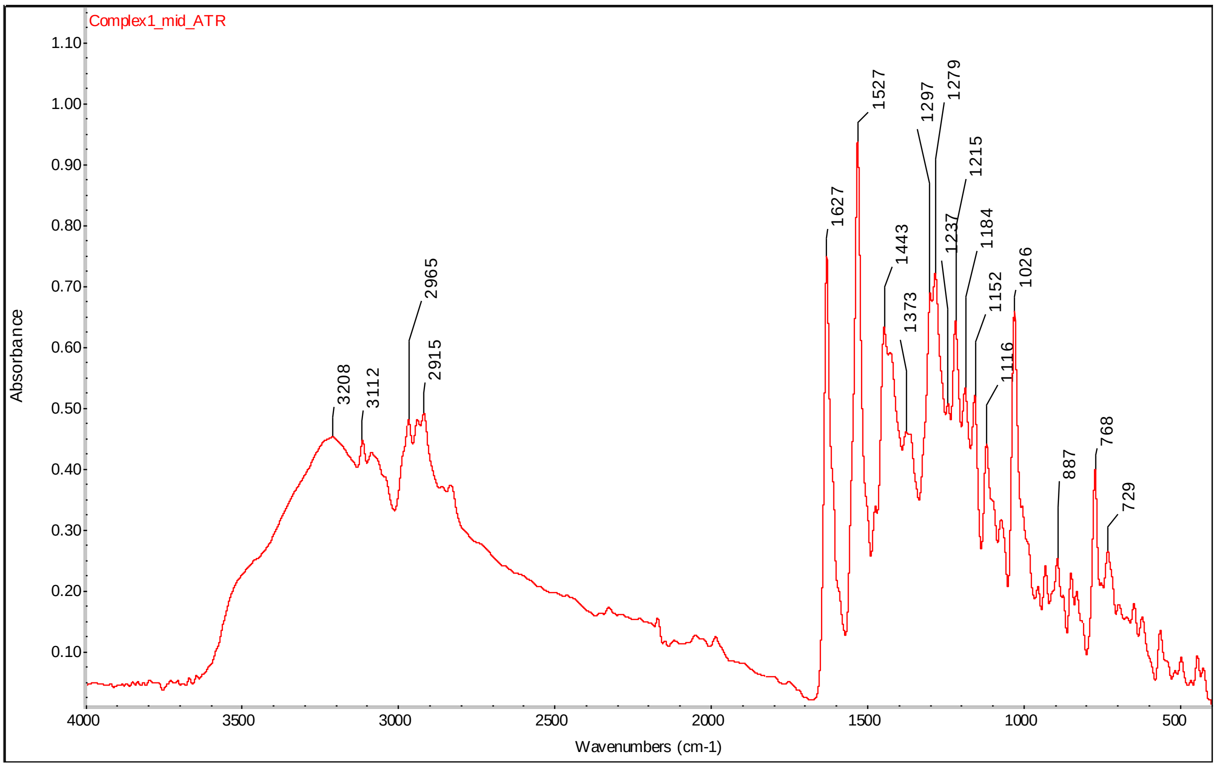

Figure S1 IR Spectrum of [Cu(L)(bpy)]NO_3·2MeOH (**1**).

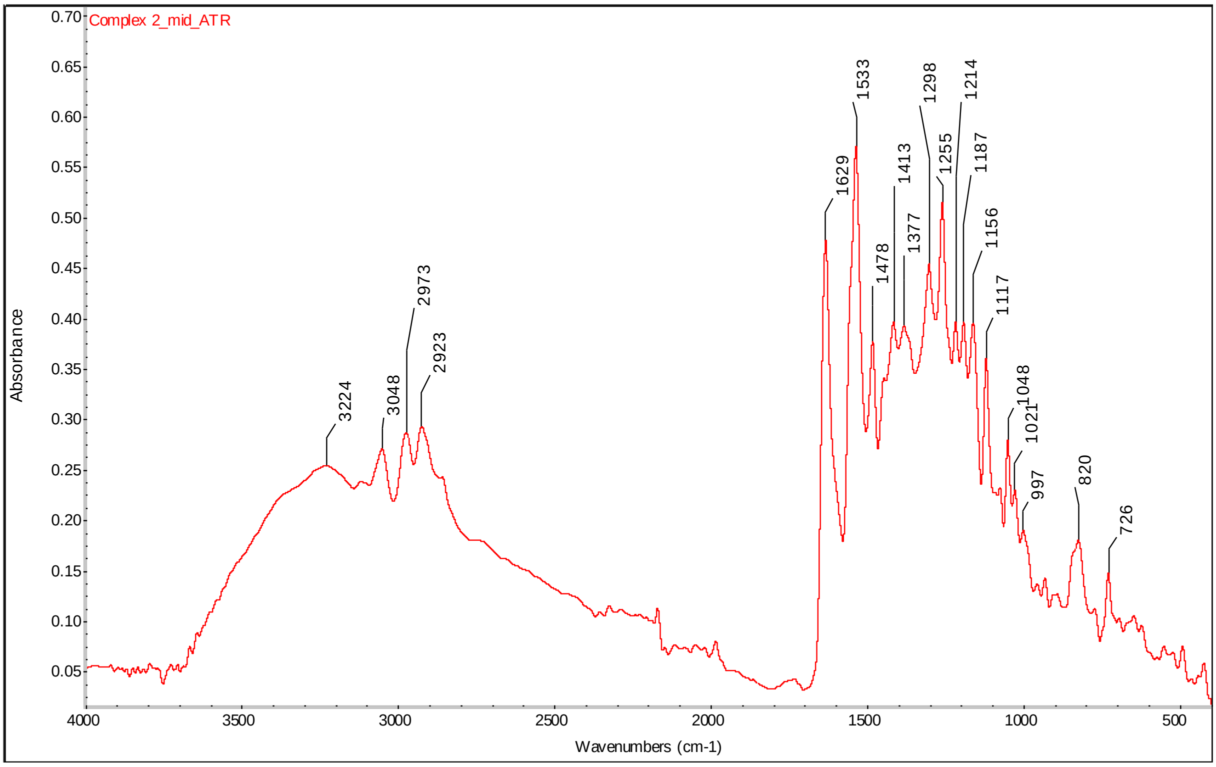

Figure S2 IR Spectrum of [Cu(L)(dimebpy)]NO_3·$2H_2O$ (**2**).

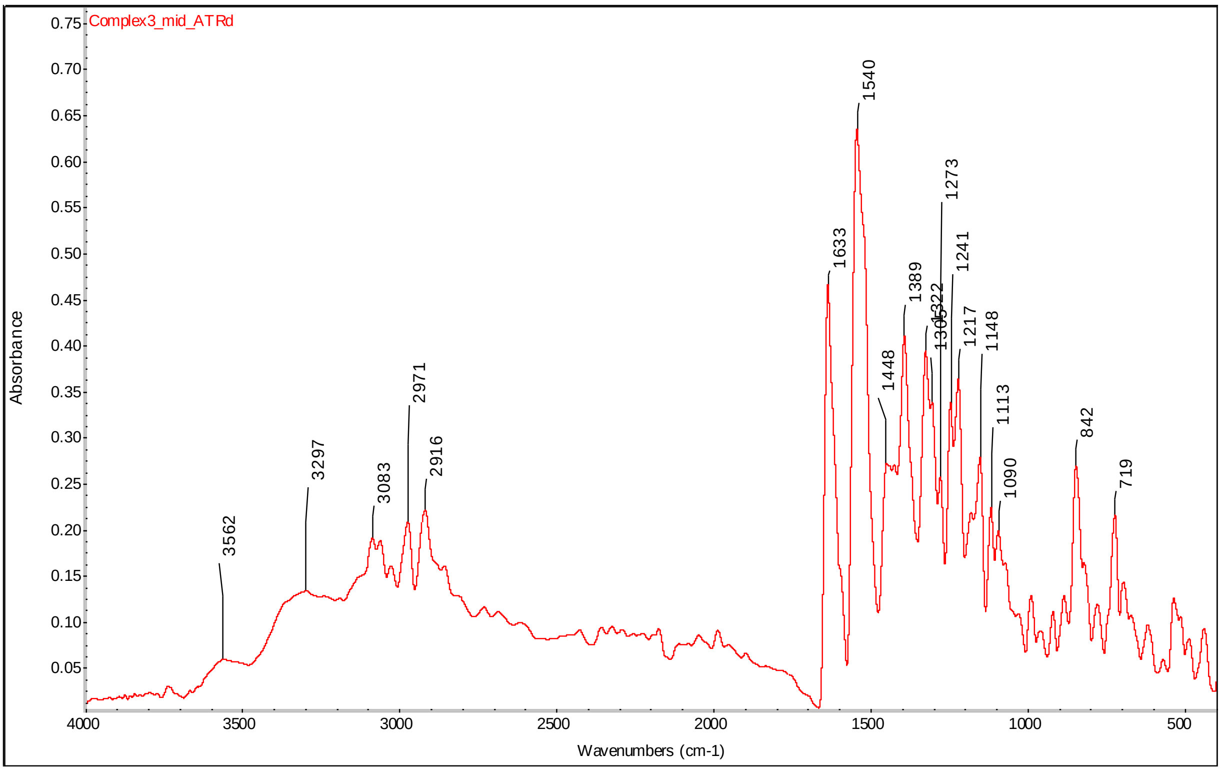

Figure S3 IR Spectrum of [Cu(L)(phen)]NO_3·2MeOH (**3**).

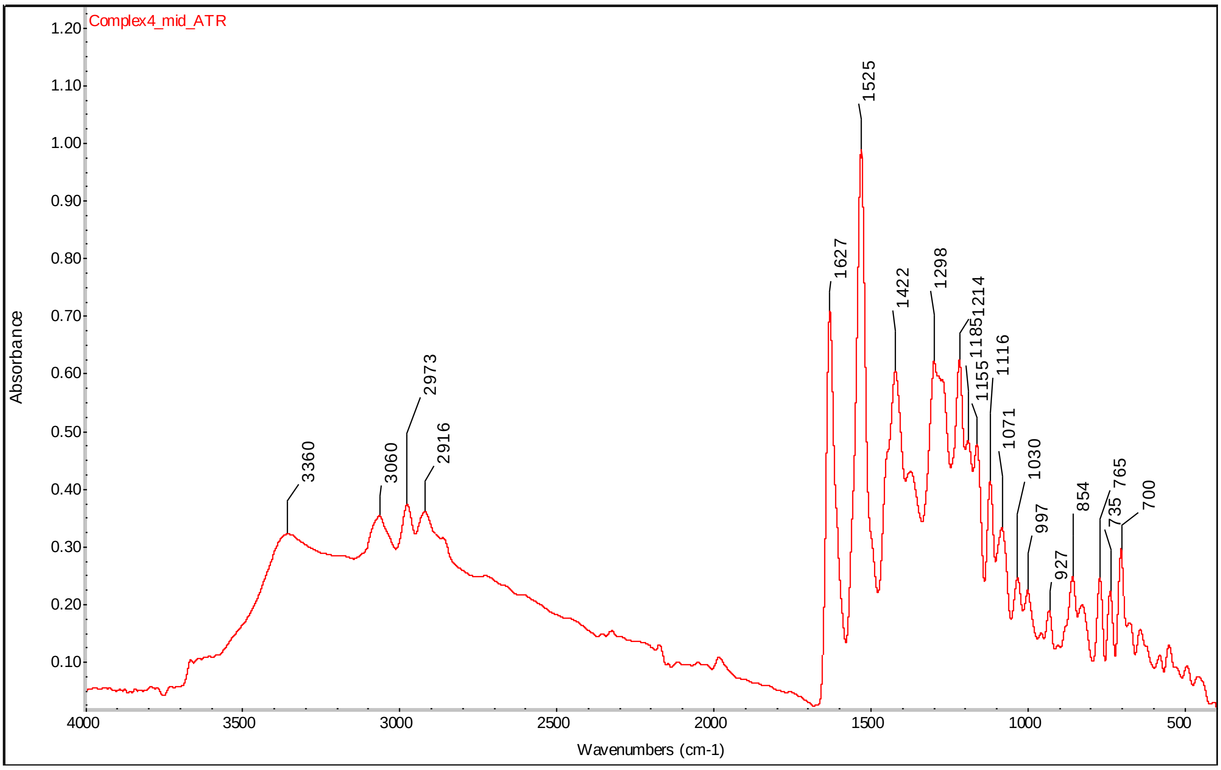

Figure S4 IR Spectrum of [Cu(L)(bphen)]NO_3·MeOH (**4**).

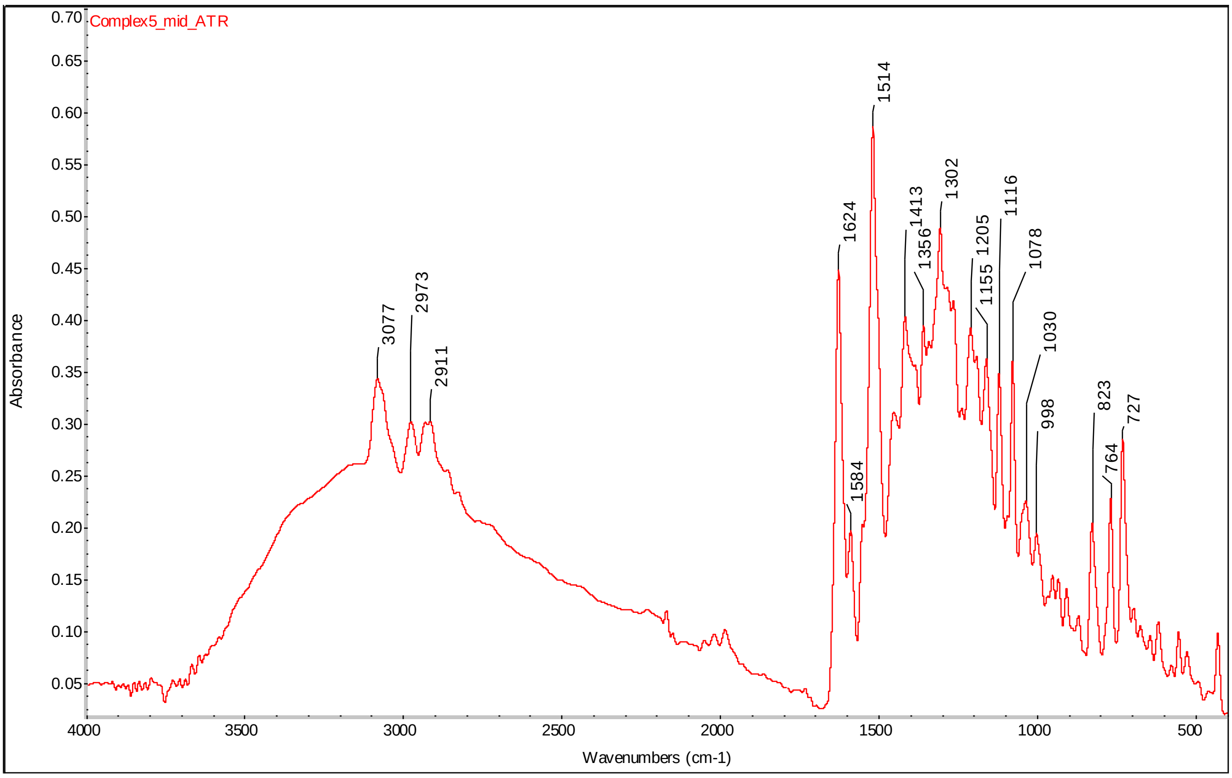

Figure S5 IR Spectrum of [Cu(L)(dppz)]NO_3·MeOH (**5**).

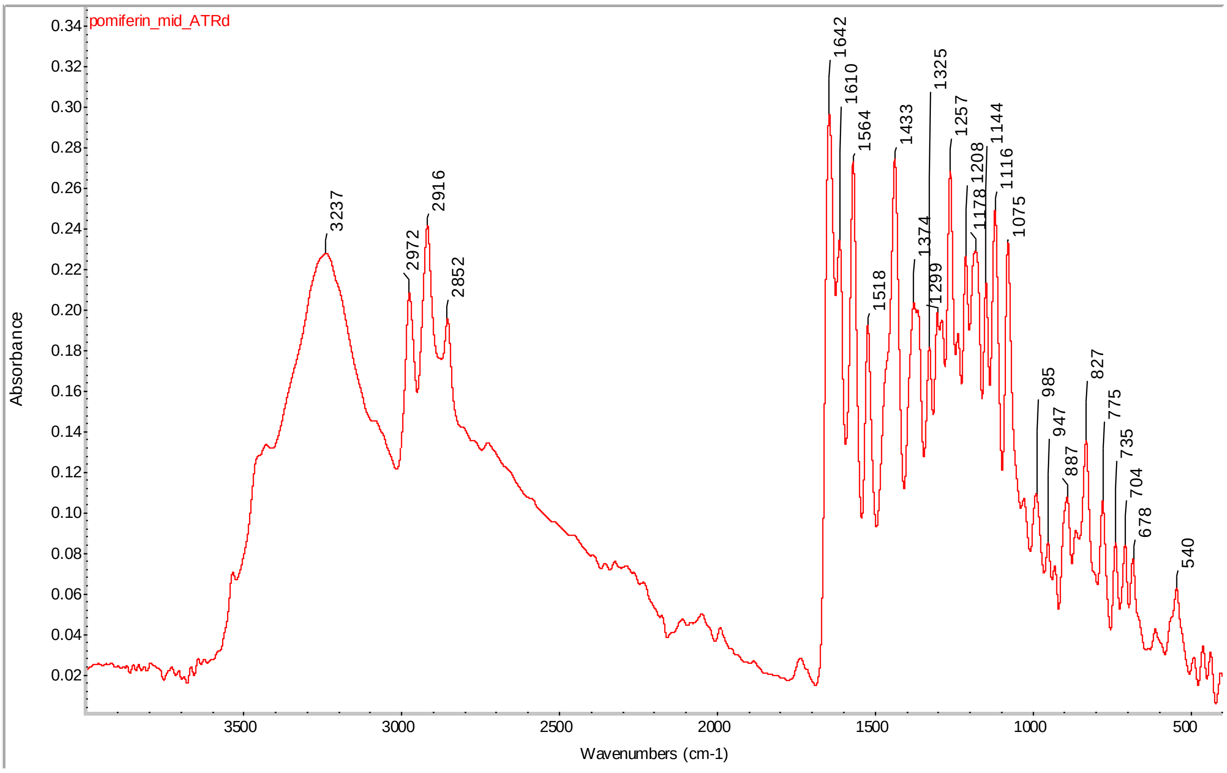

Figure S6 IR Spectrum of pomiferin (**HL**).

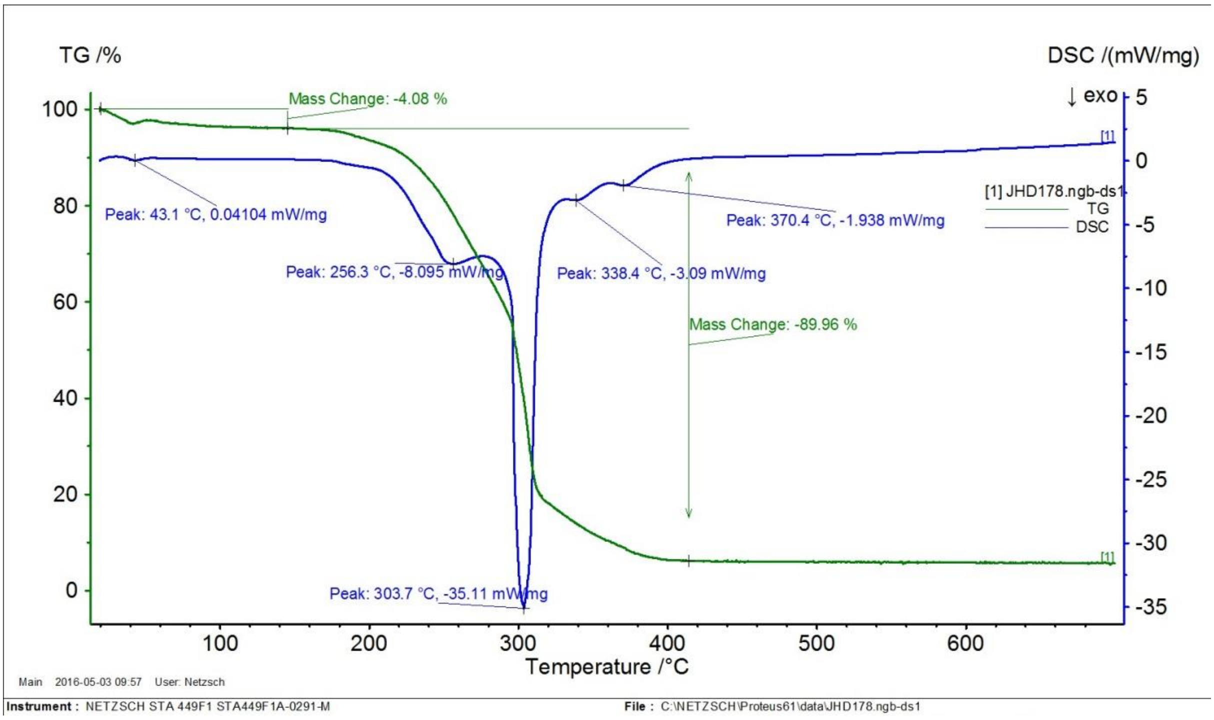

Figure S7. TG/DSC curves of [Cu(L)(bphen)]NO_3·MeOH **4** (here labelled as JHD 178). The loss of the MeOH molecule is accompanied by a theoretical weight loss of 3.52% (an experimental weight loss = 4.08%).

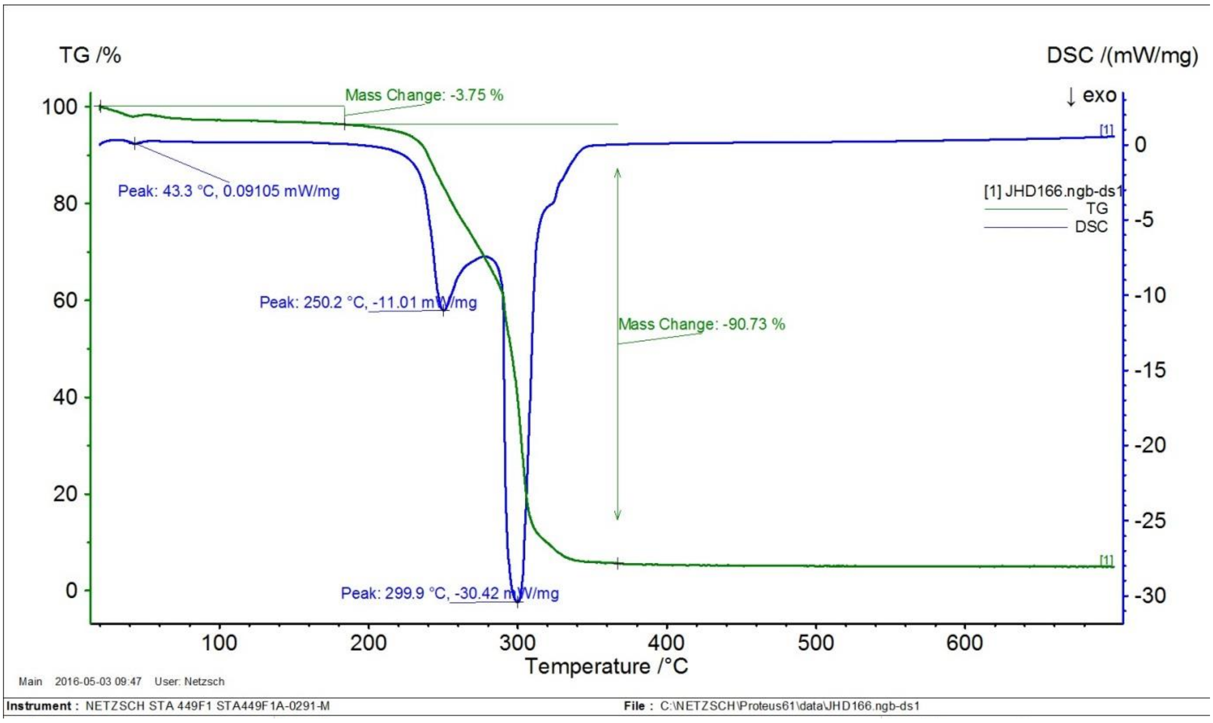

Figure S8 TG/DSC curves of [Cu(L)(dppz)]NO_3·MeOH **5** (here labelled as JHD 166). The elimination of the MeOH molecule is accompanied by a theoretical weight loss of 3.73% (an experimental weight loss = 3.75%).

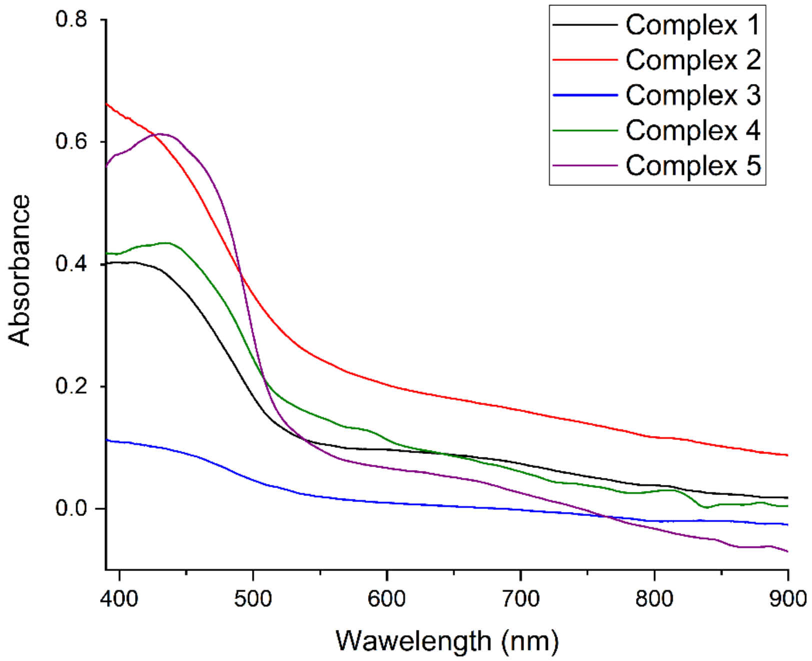

Figure S9 Diffuse reflectance UV/Vis spectra of complexes **1–5** measured in solid state (Nujol mulls).

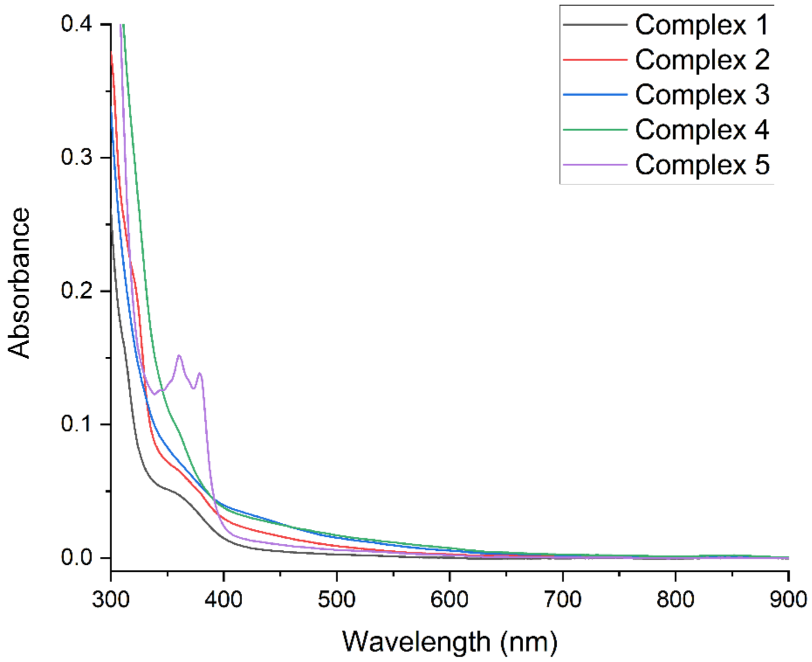

Figure S10 UV/Vis spectra of complexes **1–5** measured in DMF solutions at the 10 μM concentration.

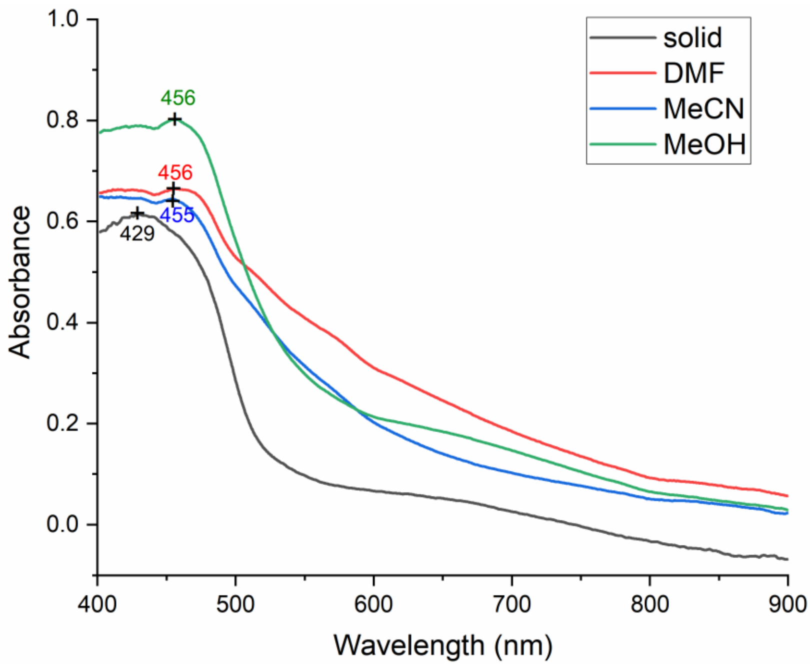

Figure S11 UV/Vis spectra of [Cu(L)(dppz)]NO_3·MeOH **5** in DMF, MeCN and MeOH (each at 15 mM concentration), and in the solid state.

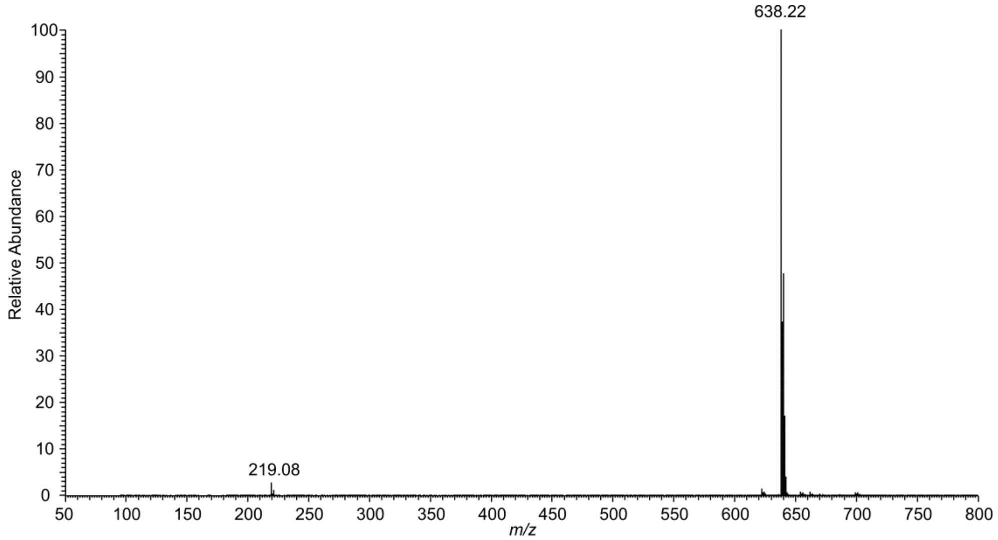

Figure S12 Mass spectra (ESI+MS) of the complex **1** showing the dominant peak associated with the corresponding complex cations.

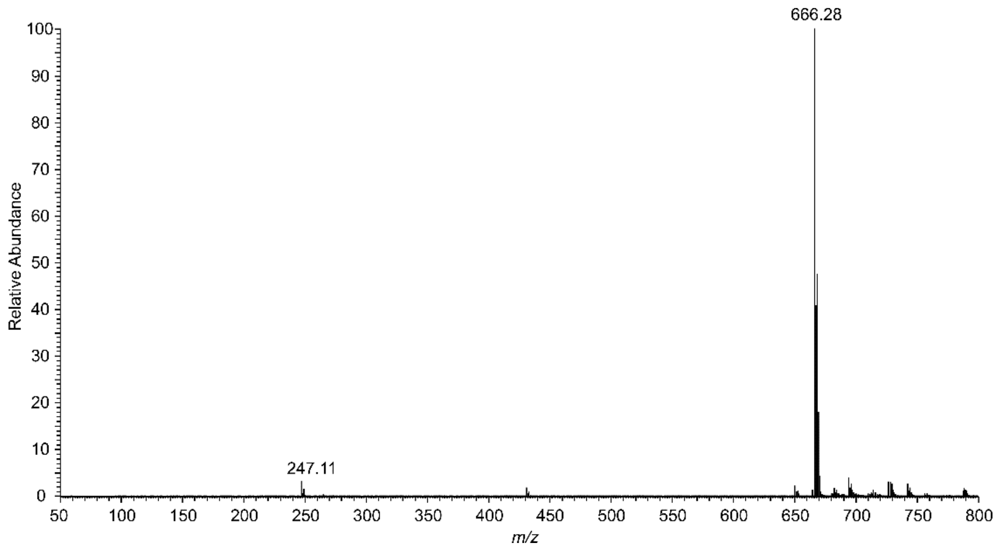

Figure S13 Mass spectra (ESI+MS) of the complex **2** showing the dominant peak associated with the corresponding complex cations.

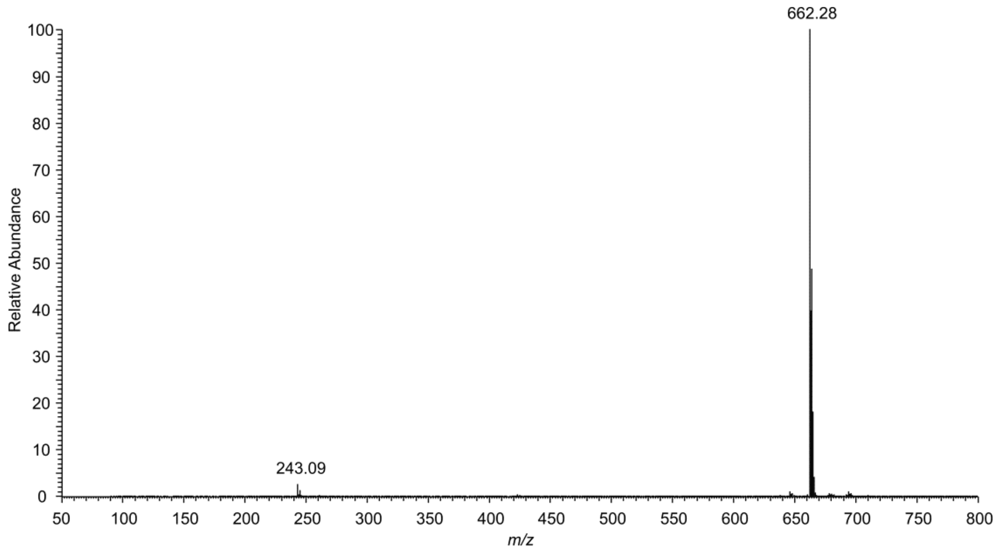

Figure S14 Mass spectra (ESI+MS) of the complex **3** showing the dominant peak associated with the corresponding complex cations.

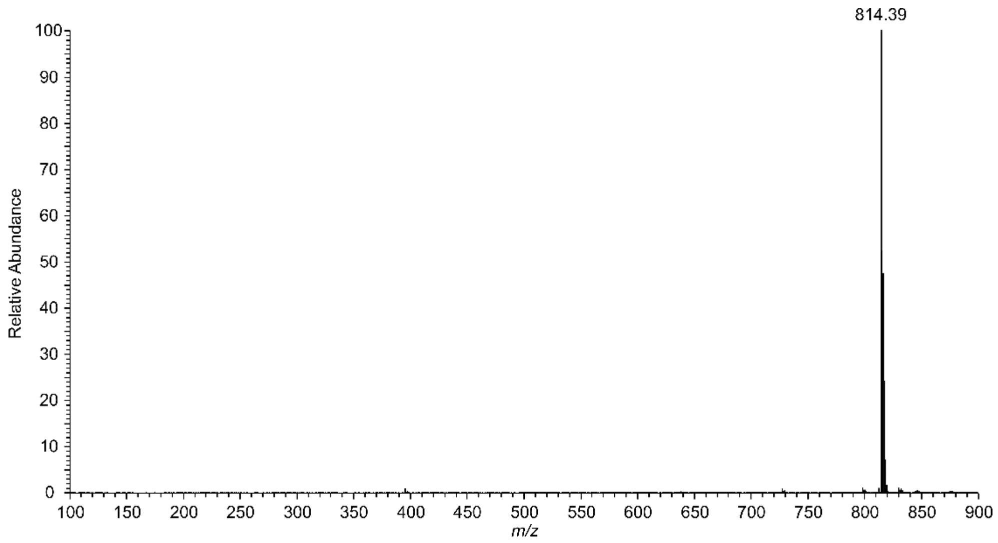

Figure S15 Mass spectra (ESI+MS) of the complex **4** showing the dominant peak associated with the corresponding complex cations.

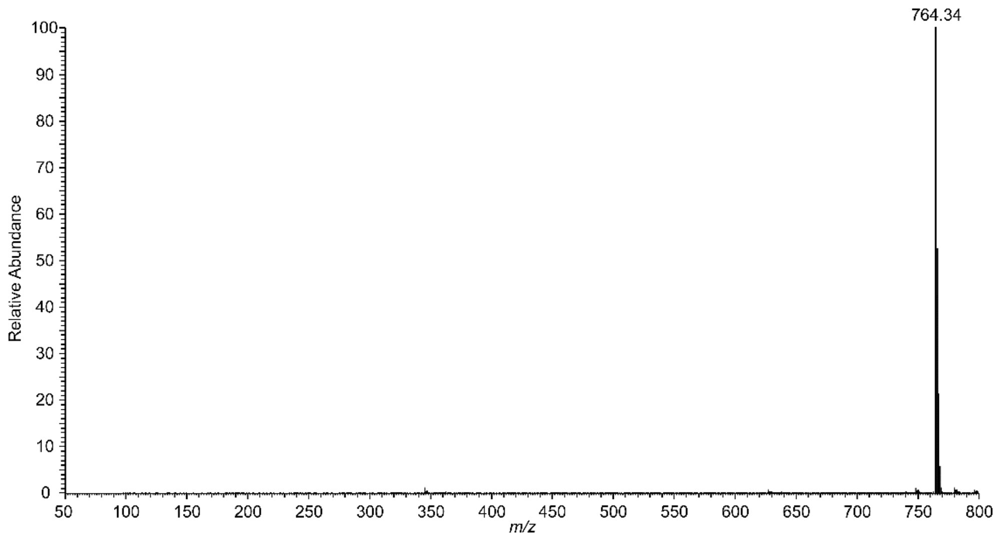

Figure S16 Mass spectra (ESI+MS) of the complex **5** showing the dominant peak associated with the corresponding complex cations.

Table S1 The values of molar conductivity of complexes **1**–**5** in different solvents.

**Complex**	Λ_M (MeCN) [S cm^2 mol^{-1}]	Λ_M (MeOH) [S cm^2 mol^{-1}]	Λ_M (DMF) [S cm^2 mol^{-1}]
**1**	143	83	68
**2**	132	84	82
**3**	143	81	85
**4**	138	81	80
**5**	n.m.	104	78
Reference data*	120-160	80-115	65-90

* Reference data for 1:1 electrolytes in the corresponding solvent, ref. W.J. Geary, The use of conductivity measurements in organic solvents for the characterization of coordination compounds, Coord. Chem. Rev. 7 (1971) 81–122. n.m. = not measured.
